# Supplementary material for: Proteome activity landscapes of tumor cell lines determine drug responses
Source: Nat Commun. 2020 Jul 20;11:3639. doi: 10.1038/s41467-020-17336-9 (PMC7371697; doi:10.1038/s41467-020-17336-9)
Supplement: Supplementary file 1 — Supplementary Information [file 41467_2020_17336_MOESM1_ESM.pdf]

Proteome activity landscapes of tumor cell lines determine drug responses

Frejno, Meng, Ruprecht et al.

SUPPLEMENTARY METHODS

Cell lines

Cell lines used in this study are part of the NCI60[1] and CRC65[2] cell line panels, as well as HeLa[3] and Jurkat cells[4]. Detailed information on these cell lines can be obtained from the cited publications, as well as from Supplementary Table 1. Cell lines were not authenticated for this study.

Breast cancer patients

The breast cancer cohort included 361 patients who received surgery between 2004 and 2012 at the Department of Gynecology of the Klinikum rechts der Isar. Informed consent was obtained from all patients with respect to scientific analyses of their data and scientific analyses of biomaterial obtained for diagnostic purposes. The mean patient age was 61 years. 316 cases were hormone receptor positive of which all were expressing the estrogen receptor (ER+) and 277 were additionally positive for the progesterone receptor (PGR+). 28 of the hormone receptor positive tumors also expressed HER2 (either 3+ by immunohistochemistry or 2+ and FISH positive; HER2+). 11 of the hormone receptor negative cases were HER2 positive and another 34 were triple-negative. For pPGR data was available for 265 out of 277 PGR positive cases. 182 cases were classified as pT1, 151 cases were pT2, 13 cases were pT3 and 15 cases were categorized as being pT4. 224 cases were node negative and 137 cases were node positive. 44 cases were graded as G1, 172 cases were G3 and 145 cases were G3, respectively. 41 patients died during follow-up. Mean follow-up time for patients still alive at the endpoint of analysis was 55.5 months. All patients received standard adjuvant therapy after resection according to the current treatment guidelines depending on the tumor’s stage and biology (chemotherapy), hormone receptor and HER2 status (targeted therapy). Briefly, ER+ and ER+/PGR+ patients received endocrine therapy (Tamoxifen or an aromatase inhibitor)

+/- chemotherapy, while HER2+ patients received Trastuzumab + chemotherapy + endocrine therapy if they were ER+ or ER+/PGR+. Triple-negative patients received chemotherapy.

AML Patients

Patients were admitted to the University Hospital Frankfurt between 2010 and 2014 and treated for newly diagnosed AML with regimens containing standard dose Cytarabine and Daunorubicin ("7+3"). Patients at the University Hospital Frankfurt are routinely advised to undergo a bone marrow biopsy at diagnosis. All patients consented to the scientific analyses of their data and to scientific analyses of biomaterial that was obtained for diagnostic purposes. All patients received at least one course of Cytarabine at a dose of 100 mg/m^2 over 7 days and Daunorubicin at a dose of 60 mg/m^2 over 3 days ("7+3") (if not stated otherwise). All patients under the age of 60 received a second cycle of induction therapy. Patients over the age of 60 received a second induction cycle only if their day-15 bone marrow aspirate showed more than 5% blasts. For the analyses, patient records were reviewed by physicians who were unaware of the AK1 expression results in the diagnostic biopsies. Remission criteria and cytogenetic risk groups were assessed according to the ELN guidelines. The initial response to induction therapy was analyzed in bone marrow biopsies and aspirates and defined as complete (CR) if the blast count was <5%, and as "no CR" if the blast count was >5%. For the calculation of event-free survival (EFS), events were defined as failure to achieve complete remission (CR, CRi, CRp) within 40 days after the last induction cycle, relapse or death at any time after start of therapy.

Cell culture

In line with the standard protocol for the *in vitro* cancer screen of the Developmental Therapeutics Program (DTP) of the National Cancer Institute, NCI60 cell lines were cultured in Roswell Park Memorial Institute (RPMI) 1640 medium containing stable glutamine and 5% fetal bovine serum (FBS; Biochrom) at 37°C and 5% CO_2. CRC65 cell lines were grown in high glucose Dulbecco's modified Eagle medium (DMEM,

including GlutaMAX and pyruvate; PAA) containing 1% Pen-Strep (penicillin at 100 units/mL and streptomycin at 100 μg/mL final concentration; PAA) and 10% FBS at 37°C and 10% CO_2. HeLa and Jurkat cell lines were cultured in Iscove's Modified Dulbecco's Medium (IMDM; #FG0465, Biochrom) including stable glutamine and 10% FBS (#S0615, Biochrom) at 37°C and 5% CO_2.

Cell lysis

Adherent cells were harvested at ~80–90% confluence. Suspension cells were harvested by centrifugation in 250-mL centrifuge tubes at 300 ×*g* and 4°C for 5 min. After two washes with PBS (without Mg^{2+} and Ca^{2+}), lysis buffer is added to each T175 cell culture flask (adherent cells) or cell pellet (suspension cells). NCI60 cells were lysed in 8 M urea, 40 mM Tris-HCl pH = 7.6 containing protease (Complete™ mini with EDTA; Roche) and phosphatase inhibitors (Phosphatase Inhibitor cocktail 1 and 2; Sigma Aldrich) at 1× and 5× of the final concentration recommended by the manufacturer. For CRC65 cells, we used RIPA100 buffer (20 mM Tris–HCl pH 7.5, 1 mM EDTA, 100 mM NaCl, 1% Triton X-100, 0.5% sodium deoxycholate and 0.1% SDS) containing protease (Complete™ mini with EDTA; Roche) and phosphatase inhibitors (Phosphatase Inhibitor cocktail 1 and 2; Sigma Aldrich) at 2× and 5× the final concentration recommended by the manufacturer. After incubating the cells for 15 min/30 min (NCI60/CRC65) on ice, total cell lysates were cleared by centrifugation at 22,000 ×*g* for 60 min/30 min (NCI60/CRC65) at 4°C. The pellet was discarded and the protein concentration of the supernatant was determined using a Bradford assay (Pierce).

Acetone precipitation and re-solubilization

Total cell lysates of the CRC65 cell line panel were acetone-precipitated and re-suspended in urea buffer (40 mM Tris/HCl pH = 7.6, 8 M urea) containing protease (Complete mini without EDTA; Roche) and phosphatase inhibitors (Phosphatase Inhibitor cocktail 1 and 2; Sigma Aldrich) at 1× and 5× the final

concentration recommended by the manufacturer, respectively, as well as 20 nM calyculin A as described previously[2].

Enzymatic Digestion

For in-solution digestion, we used 2 mg/3.5 mg of protein per cell line of the NCI60/CRC65 cell line panel, respectively. To reduce disulfide bonds, DTT was added to a final concentration of 10 mM followed by incubation for 40 min at room temperature on a thermoshaker at 700 rpm. Alkylation of cysteine residues was achieved by addition of chloroacetamide to a final concentration of 55 mM and incubation for 20 min at room temperature in the dark. Samples were diluted to a final concentration of 1.5 M urea by addition of 40 mM Tris–HCl pH 7.6. 1 mM $CaCl_2$ was added to each sample to improve trypsin digestion. Subsequently, proteins were digested overnight at 37°C and 700 rpm in a thermomixer using sequencing grade trypsin (Promega for NCI60; Roche for CRC65) or Glu-C (Promega) at a protease-to-protein ratio of 1:50 (w/w). A second batch of trypsin was added to trypsin digests of the NCI60 panel at 1:50 protease-to-protein ratio (w/w) after 4 h and digestion was continued overnight.

The next day, the peptide digest was acidified using TFA to reach a final pH of 2-3. After centrifugation for 5 min at 5,000 ×*g*, desalting of the peptide mixture supernatant was achieved using Sep-Pak cartridges (50 mg sorbent per cartridge; Waters) and a vacuum manifold according to the manufacturer's instructions. The SepPak eluate was adjusted to an ACN concentration of 30% and directly used for phosphopeptide enrichment.

Phosphopeptide enrichment using Fe-IMAC

Phosphopeptide enrichment was essentially performed as previously described using 2 mg and 3 mg of protein digest for the NCI60[5] and CRC65[6] panel, respectively. Briefly, we used a Fe-IMAC column (ProPac IMAC-10 column, 4 × 50 mm, Thermo Fisher Scientific) connected to an Aekta HPLC system, which was initially charged with $FeCl_3$ and equilibrated with IMAC solvent A (30% ACN, 0.07% TFA). Afterwards,

Sep-Pak-eluted peptides adjusted to a final concentration of 30% ACN were loaded onto the column. Subsequently, peptides were eluted in a step-wise gradient from 0% to 11.5% solvent B (0.3% NH_4OH) in 5 min and 11.5% to 26% solvent B in 19 min. A UV chromatogram at 214 nm and 280 nm was recorded for each enrichment run. Both the flow-through and the phosphopeptide elution fraction were collected, dried down and stored at -80°C.

High pH reversed-phase fractionation

High-pH reversed-phase (RP) tip fractionation of full proteome (NCI60) and phosphoproteome digests (NCI60 & CRC65) was essentially performed as previously described[7]. We used 50 µg dried full proteome digest and the entire phosphorylated fraction of peptides for the NCI60 panel (2 mg starting material) or one third of it for the CRC65 panel (3 mg starting material). Briefly, 200 µL pipette tips were packed with five C-18 extraction disks (Ø 1.5 mm, 3M Empore) and fixed in 1.5 mL microcentrifuge tubes. All solvents were passed through the tip by centrifugation (800 ×*g*, room temperature). First, tips were primed using 250 µL of 100% ACN, followed by 250 µL of 50% ACN in 25 mM NH_4COOH, pH 10 and 2 × 250 µL of 25 mM NH_4COOH, pH 10. Next, dried phosphopeptides or full proteome digest were re-solubilized in 250 µL of 25 mM NH4COOH, pH 10 and loaded onto the C18 material. After re-application of the flow through, phosphopeptides were eluted using increasing concentrations of ACN (2.5% / 7.5% / 12. 5% / 50% ACN in 25 mM NH_4COOH). The previously stored flow through, which was desalted according to Rappsilber et al.[8], was combined with the 50% ACN eluate, resulting in four fractions. Non-phosphorylated peptides were eluted using 5%, 10%, 15%, 20% and 50% ACN. Flow through and 20%, as well as 5% and 50% fractions of each high pH-RP fraction were injected per full proteome/phosphoproteome sample (to avoid potential iron contamination, 50 mM citrate was added to the reconstitution solvent used for the phosphopeptide fraction).

Quality control of the enrichment workflow

As a means of quality control, a library of 60 phosphopeptides and their corresponding non-phosphorylated counterpart sequences (for a total of 120 peptides) were spiked into each batch of cell line lysate from the NCI60 panel prior to enzymatic digestion. To reach sufficient quantities for all 120 batches (NCI60 trypsin and Glu-C digests), we combined sixteen individual wells B2 of the phosphopeptide library synthesized by Marx et al.[9]. After desalting the pooled peptides using STAGETips[8], the dried down eluates were reconstituted in 800 µL of 0.1% FA. We added 5 µL reconstituted spike-in peptides to each batch of cell line lysate. In addition to the synthetic phosphopeptides, we controlled our phosphopeptide enrichment workflow with HeLa cells, which were processed in the same way as the cell lines from the NCI60 and CRC65 cell line panels. They were included in the MaxQuant searches of our phosphoproteome data. We also measured the phosphoproteome of HeLa cells without high pH RP fractionation and compared the quantification of (spike-in) p-sites between the two workflows in order to make sure that high pH RP fractionation does not negatively affect our results (see Supplementary Figure 1C-D).

LC-MS/MS data acquisition

Peptides were delivered to a trap column (75 µm × 2 cm, packed in-house with 5 µm C-18 resin; Reprosil PUR AQ, Dr. Maisch) and washed using 0.1% formic acid at a flow rate of 5 µL/min for 10 min. Subsequently, peptides were transferred to an analytical column (75 µm × 45 cm, packed in-house with 3 µm C-18 resin; Reprosil Gold, Dr. Maisch) and separated using a linear or stepwise gradient from LC solvent A (0.1% FA in 5% DMSO[10]) to LC solvent B (0.1% FA, 5% DMSO in ACN) at a flow rate of 300 nL/min. The exact gradient composition was dependent on the sample type: trypsin/Glu-C full proteome digest fractions were separated in a linear gradient from 0% solvent B to 34% solvent B in 111 min. Phosphopeptide fractions were separated in a step-wise gradient from 0% solvent B to 15% solvent B in 69 min followed by an increase to 27% solvent B in 30 min.

The mass spectrometer was operated in positive ionization mode using data dependent acquisition, automatically switching between MS1 and MS2 spectra. Full scan MS1 spectra were recorded from 360 m/z to 1300 m/z at a resolution of 60,000 (at 200 m/z) using an AGC target value of 3e6 charges and a maximum injection time of 10 ms. Up to 20 (12 for phosphopeptide samples) sequentially selected precursors (isolation window 1.7 m/z) were fragmented via HCD using a normalized collision energy of 25%. For full proteome digests, MS2 spectra were recorded at a resolution of 15,000 using an AGC target value of 1e5 (2e5 for Glu-C digests) and a maximum injection time of 25 ms (50 ms for Glu-C digests). MS2 spectra for phosphoproteome digests were recorded at a resolution of 30,000 with an AGC of 2e5 charges at a maximum injection time of 120 msec. All measurements had a fixed first mass set to 100 m/z and a dynamic exclusion of 30 sec.

Cell viability assays

In order to test our sensitivity predictions for 5FU and our activity-driven hypothesis that inhibitors targeting MET and MST1R can act synergistically in cell lines with high phosphorylation of these receptor tyrosine kinases, we performed *in-vitro* cell viability assays. These assays were performed as described previously[2], with minor modifications. Briefly, we generated 10-point dose-response curves in technical and biological triplicates for single-compound and combination treatments (constant mixing ratios) in a 96-well format. For 5FU (#1209, Selleckchem), we selected two cell lines predicted to be sensitive (RPMI8226, HCC2998) and two cell lines predicted to be resistant to the drug (CCRFCEM, KM12), making sure to include representatives in both categories for AML and CRC, respectively. For single-compound and combination treatments with drugs targeting MET and MST1R, we selected Tepotinib (#7067, Selleckchem; MET inhibitor) and MK-2461 (#D2650, Sigma Aldrich; MST1R inhibitor) based on a recent large-scale study investigating the target landscape of kinase inhibitors[11] and decided to treat HDC-8 cells based on their high phosphorylation of these receptor tyrosine kinases. On day zero, $5x10^4$ cells were

seeded in 100 µL IMDM (#FG0465, Biochrom; RPMI8226, HCC2998, CCRFCEM and KM12) or DMEM (#FG0445, Biochrom; HDC-8) including stable glutamine and 10% FBS (#S0615, Biochrom). After incubating the cells overnight under standard cell culture conditions (37°C and 5% CO_2), 50 µL of fresh medium containing either the respective drug or an equivalent amount of DMSO (#D2650, Sigma Aldrich) were added, resulting in one of ten different final drug concentrations (5FU: 30 µM, 10 µM, 3 µM, 1 µM, 0.3 µM, 0.1 µM, 0.03 µM, 0.01 µM, 0.003 µM, 0 µM; Tepotinib: 1 µM, 0.3 µM, 0.1 µM, 0.03 µM, 0.01 µM, 0.003 µM, 0.001 µM, 0.0003 µM, 0.0001 µM, 0 µM; MK-2461: 100 µM, 30 µM, 10 µM, 3 µM, 1 µM, 0.3 µM, 0.1 µM, 0.03 µM, 0.01 µM, 0 µM). For combination treatments with Tepotinib and MK-2461, the drugs were combined at constant ratios over the entire concentration range used for single agent treatments (1:100 Tepotinib:MK-2461). Following 96h of incubation under standard cell culture conditions, 15 µL AlamarBlue (#DAL1100, Thermo Fisher Scientific) were added and after additional 3h of incubation at 37°C and 5% CO_2 AlamarBlue fluorescence was quantified using a FLUOstar Omega plate reader (BMG Labtech).

Immunostaining of breast cancer samples

Immunohistochemical (IHC) staining of TMAs for PGR was part of routine diagnostic pathology. In addition, we performed triplicate staining of TMAs for PGR_pS162 for each patient. Immunohistochemistry was carried out on a BenchMark XT automated slide staining instrument (Ventana, Tucson, AZ) using a PGR (#PI633C01, clone 16, DCS) and a PGR_pS162 antibody (#GTX80351-50, Lot Nr. 821604961, Biozol) together with the ultraVIEW DAB Detection Kit (#760-500; all reagents from Ventana, Tucson, AZ). Briefly, the tissue sections were deparaffinized with EZ Prep at 75°C and 76°C, heat pretreated in Cell Conditioning 1 (CC1) for antigen retrieval at 76°C – 100°C and then incubated with the primary antibody diluted in antibody diluent at 1:50 (PGR) or 1:100 (PGR_pS162) for 32 min at 37°C after inactivation of the endogenous peroxidase activity using UV-inhibitor for 4 min at 37°C. Subsequently, the slides were incubated with an HRP Universal Multimer for 8 min. Antibody binding was

detected using DAB as chromogen, followed by counterstaining with hematoxylin for 10 min with subsequent bluing in bluing reagent for 10 min. Slides were then dehydrated manually by alcohol washes of increasing concentration (70%, 96%, 100%) and xylene and finally coverslipped using Pertex® mounting medium (#00801, Histolab, Goteborg, Sweden). A board-certified pathologist with a special expertise in breast pathology (ED) evaluated the slides. Based on the PGR classification rules used in routine diagnostic pathology, a case was deemed PGR positive if any nuclear staining was seen, regardless of the strength of staining and the percentage of cells stained. For PGR_pS162, we scored both intensity of staining (none=0, weak=1, moderate=2, strong=3) as well as percentage of positive cells (from 0-100%). From both parameters, we calculated modified H-scores (0-300)[12] for each patient by combining the IHC triplicates in the following way:

$$h = \frac{1}{3}(s_1 \cdot p_1 + s_2 \cdot p_2 + s_3 \cdot p_3) \tag{1}$$

Here, s_i is the staining intensity of replicate i and p_i is the percentage of stained cells of replicate i. We defined PGR_pS162 H-scores < 110 as pPGR=low and PGR_pS162 H-scores >= 110 as pPGR=high.

Immunostaining of bone marrow samples

Bone marrow samples from 79 AML patients were provided by the University Hospital Frankfurt, Germany. Tissues were fixed in 4% buffered formalin, decalcified in EDTA and embedded in paraffin. Immunohistochemical staining was performed as previously described[13]. Briefly, standardized pretreatment steps included deparaffinization, rehydration, and unmasking Trilogy (Cell Marque, USA) for 12 min. Subsequent staining included primary antibodies directed against adenylate kinase 1 (Anti-AK1, #OAAB17548, Aviva Systems Biology, USA) with a dilution of 1:50 and incubation time of 45 min. AK1 KO cells were used as a negative control. Visualization was performed after applying a polymer kit (ZytoChem Fast AP, Zytomed Systems, Germany) with a Fast Red Substrate Kit (Zytomed System, Germany) and counterstaining with Meyer's hematoxylin (#K8008, DAKO).

Two pathologists, who were blinded to clinical history and therapeutic response, independently scored the AK1 IHCs. They evaluated all tissue sections for positive staining using a four-stage staining score: 0 = negative; 1 = weak intensity in < 25% of blasts; 2 = weak intensity in ≥ 25% of blasts; 3 = strong intensity in ≥ 25% of blasts. IHC staining scores of 0 and 1 were defined as no or low expression and IHC staining scores of 2 and 3 were defined as high AK1 expression.

In-vitro dephosphorylation of antimetabolites

Antimetabolite dephosphorylation assays were performed as described earlier[14], with minor modifications. Briefly, we prepared reaction mixtures (15 µL) using a Tris-based buffer (50 mM Tris-HCl pH 7.5, 10 mM $MgCl_2$, 100 mM NaCl, 1 mM DTT), which contained 1 mM AMP (#01930, Sigma Aldrich) as phospho-acceptor and either 1 mM ATP (#A6419, Sigma Aldrich; positive control) or 1 mM of the tri-phosphorylated version of Cytarabine (ara-CTP; #NU-1170S, Jena Bioscience), Nelarabine (ara-GTP; #N-1100, Trilink Biotechnologies), Zalcitabine (ddCTP; #N-4005, Trilink Biotechnologies) or Gemcitabine (dfCTP; #NU-1607S, Jena Bioscience) as phospho-donor. Reactions were started upon the addition of 100 µg recombinant AK1 (#ab167983, Abcam) or an equivalent volume of buffer (mock) to the mixture. We incubated the reactions at 37°C and stopped them after 30 min by adding 15 µL pure ACN to the mixture in order to precipitate the recombinant protein. Afterwards, the samples were centrifuged for 20 min at 4°C and 20,000 ×*g* and the supernatants were stored on ice until they were measured by LC-MS/MS.

MRM of ATP and antimetabolites

AK1 reaction mixtures were subjected to LC-MS/MS analysis using a Prominence HPLC system (Shimadzu, Germany) and a 4000 QTRAP (Applied Biosystems, Forster City, USA). The separation was performed using a 150 x 2.1 mm inner diameter, 3.5 µm xBridge Amide HILIC column (Waters, Eschborn, Germany) with a flowrate of 400 µL/min. We used a linear gradient from 10% A (A: 5 mM ammonium

acetate, pH 9.5, B: acetonitrile/water with 5 mM ammonium acetate in a 95/5 v/v mixture, pH 9.5) for 1 min to 100% A in 10 min, which was maintained for 5 min. Afterwards, the column was equilibrated to starting conditions. Ions were analyzed by MS in negative mode using a Turbo V Ion Source (Applied Biosystems). The ion spray voltage was set to -4500 V at a source temperature of 450°C using nitrogen as collision gas. The parameters for collision-activated dissociation (CAD) were: medium; curtain gas: 35 psi; ion source gas 1: 55 psi; ion source gas 2: 65 psi. Multiple reaction monitoring (MRM) conditions for ATP and (phosphorylated) antimetabolites are listed in Supplementary Table 11.

Overexpression of AK1 in Jurkat cells

In order to test our hypothesis that AK1 can dephosphorylate and thereby inactivate Cytarabine, we overexpressed AK1 in Cytarabine-sensitive Jurkat cells (as evaluated using ProteomicsDB[15]) and quantified the fraction of apoptotic cells over time using live-cell imaging in a 96-well format. We performed the assay in technical triplicates and repeated it thrice (biological triplicates). On day zero, we coated the 96-well plate with 50 µL Poly-L-Lysine (#L7240, Biochrom) for 45 min to ensure adherence of Jurkat cells. After washing the wells twice with 100 µL PBS (#D8662, Sigma Aldrich), $2x10^5$ cells were seeded in 50 µL IMDM with stable glutamine and 10% FBS, followed by the addition of 50 µL Interferin-based (#409-10, Polyplus) transfection mix in OptiMem (#31985-062, Life Technologies) with or without AK1 expression vector (#RC515130, Origene) at a final concentration of 1 nM (prepared according to the manufacturer's instructions). From then on, plates were incubated at 37°C and 5% CO_2 in an IncuCyte S3 live-cell analysis system (IncuCyte), which monitored their confluence in the phase-contrast and apoptosis in the red channel every 2 h (excitation at 630 nm and emission at 650 nm; apoptosis reagent only added later). After 72 h of incubation, 50 µL IMDM containing stable glutamine and 10% FBS, as well as Caspase-3/7 reagent at a final concentration of 160 nM (#4704, IncuCyte) and Cytarabine (#S1648, Selleckchem) at one of four different final concentrations (1 µM, 0.3 µM, 0.1 µM, 0.03 µM) was added and the cells were incubated for additional 48 h at 37°C and 5% CO_2. AK1 overexpression was verified five days post transfection by

western blot using an anti-AK1 antibody (#OAAB17548, Aviva Systems Biology, USA) together with an anti-GAPDH antibody (#sc-365062, Santa Cruz Biotechnology, USA) to control for equal loading and employing standard protocols.

Raw data processing

Peptide and protein identification and quantification was performed using MaxQuant (version 1.5.5.1)[16] by searching the MS2 data against all canonical protein sequences as annotated in the UniProt reference database (42,118 entries containing canonical human protein sequences/isoforms and spike-in phosphopeptide library sequences[9]; downloaded on 14th of November 2016) using the embedded search engine Andromeda[16]. Carbamidomethylated cysteine was set as a fixed modification and phosphorylation of serine, threonine, and tyrosine, oxidation of methionine, and N-terminal protein acetylation were allowed as variable modifications. Depending on the dataset, Trypsin/P or Glu-C was specified as the proteolytic enzyme and up to two missed cleavages were allowed. Precursor and fragment ion tolerances were 10 ppm and 20 ppm, respectively. We enabled iBAQ quantification only for full proteome data and used match-between-runs for all analyses. The alignment time window was set to 20 min with a matching time window of 1 min. Search results were filtered for 1% PSM, peptide and protein FDR and a minimum peptide length of seven amino acids. We additionally excluded all modified peptides with Andromeda scores lower than 40. Phosphorylation sites (p-sites) with a localization probability > 0.75 were treated as class I sites (Supplementary Figure 1E). Raw files of CRC65 full proteomes, acquired by Frejno et al.[2] were downloaded and reprocessed using the exact same parameters as mentioned above.

Data post-processing and filtering

If not stated otherwise, we used the proteinGroups.txt and Phospho (STY)Sites.txt tables of the MaxQuant output for all our full proteome and phosphoproteome analyses, respectively. The abundance of proteins and p-sites was estimated using intensity-based absolute quantification (iBAQ)[17] and MS1 precursor

intensities (denoted as AU throughout this study). Reverse sequences in both datasets were excluded, followed by median-centering of all samples to the overall median of the respective dataset. Missing values in full proteome and phosphoproteome data were imputed using the protein-wise half-lowest method (analogous to the LOD2 method[18]) based on the rationale that the missing values in mass spectrometry experiments tend to accumulate at the lower end of the overall intensity distribution. Subsequently, the data were $\log_{10}$ transformed. If not stated otherwise, all comparisons throughout this manuscript are relative. If we state that a protein/phosphoprotein/p-site is high in a certain cell line, then it means that it’s abundance is higher compared to other cell lines without specifying any further metric or scale.

In addition to our p-site data, we also rolled-up MS1-based phosphopeptide intensities to form phosphoprotein intensities analogous to what is commonly done for peptides to calculate protein intensities and acknowledging the limitations of this approach. Here, it is worth keeping in mind that the phosphorylation of different sites on the same protein is regulated by different kinases and phosphatases and can result in different effects on cellular signaling (e.g. activating versus inactivating p-sites). To do so, we used MaxQuant’s relational database output and mapped each phosphopeptide and its intensity in the evidence.txt output file to the protein group from the proteinGroups.txt output file to the quantification of which the respective phosphopeptide intensity would contribute. Afterwards, we summed up all phosphopeptide intensities for each cell line and protein group to yield phosphoprotein intensities. We restricted our analysis to the phosphoprotein group with the highest mean intensity per gene name. Phosphoprotein intensities were used for outlier analyses and sparse multiple-block partial least squares regression (SMBPLSR; see the SMBPLSR section). It is worth noting that we excluded CoCM-1 cells from all analyses because of a consistently lower amount of identified proteins and p-sites (Supplementary Figure 1E).

Drug target data

We downloaded drug target data from Klaeger, Heinzlmeir, Wilhelm, et al.[11], who profiled the target landscape of 243 clinical kinase inhibitors using Kinobeads[19]. The pK_d ($-\log_{10}$-transformed K_d value) values were used without further processing.

Calculation of selectivity scores

Kinase inhibitors (KI) often target multiple kinases[11], making it difficult to discern which target kinase is responsible for a phenotype (e.g. sensitivity to a KI) in a particular cell line. We combined the (phospho)proteome landscapes presented in this study with phenotypic data and the previously published target landscape of clinical kinase drugs[11] to identify the target protein responsible for the observed phenotype. To facilitate this target deconvolution, we calculated a "selectivity score" S for every kinase/drug/cell line combination based on either median-centered protein or phosphoprotein intensities:

$$S = \frac{T_{K^P}}{O_{K^P}} \tag{2}$$

Here, T_{K^P} is the (phospho)protein intensity of the kinase of interest in cell line P if the KI of interest K targets it, or otherwise zero. O_{K^P} is the summed (phospho)protein intensity of the kinase of interest and all other kinases also inhibited by KI K in cell line P. Here, weakly binding off-targets with $K_{d^{app}}$ values lower than 1/10 of the $K_{d^{app}}$ value of the kinase of interest to KI K were excluded[11]. It is worth noting that researchers can dynamically explore the selectivity scores calculated based either on protein or phosphoprotein intensities in our ATLANTiC web application. In addition, users can examine selectivity plots in order to get a better understanding of the underlying data (see also Supplementary Figure 1F and G). Similarly, researchers can also use our web application to select an appropriate cell line model and/or KI to study the function of a particular kinase of interest. Here, one would select a cell line/KI combination with a high selectivity score for the kinase of interest.

Dose-response modelling

In this study, we integrated drug sensitivity information from six publicly available datasets: DTP[20], CTRP[21], GDSC[22], CCLE[23] and two small studies published by Medico et al.[24] and Bracht et al.[25]. The DTP data and the data by Bracht et al. (GI50 values) were downloaded from "Cellminer [https://discover.nci.nih.gov/cellminerdata/rawdata/DTP_NCI60_RAW.zip]" on 28th of March 2017 and from Supplementary Table 2 of the corresponding publication on 2nd of July 2014, respectively. While the data by Bracht et al. was used as supplied, the DTP data was only -$\log_{10}$-transformed. The remaining four datasets were downloaded and re-processed as described earlier[2]. Briefly, we normalized the dose-response data into a range between 1 (no response or full viability) and 0 (full response or no viability) and subsequently fitted the classical symmetric four-parameter log-logistic model to each combination of drugs and cell lines in each dataset:

$$f(x,(b,c,d,e)) = c + \frac{d-c}{1+\exp\left(b(\log(x)-\log(e))\right)} \tag{3}$$

We estimated the four parameters *c*="Lower Limit", *d*="Upper Limit", *b*="Slope" and *e*="ED50" from the data and subsequently extracted them from the fitted model. Finally, we computed sensitivity scores for each model as sAUC=1-AUC, where AUC is the standardized area under the dose-response curve in log-space, estimated using a modified computeAUC function from the drexplorer package v1.1.2[26] as described earlier[2]. The sAUC values from these four datasets or the -$\log_{10}$-transformed GI50 values from the DTP dataset were then used for all subsequent analyses. The compounds do not have a single experiment passed quality control[20] were excluded. We used 20,000 compounds from the DTP dataset for outlier analyses but restricted this dataset to a subset of drugs with either a known mode-of-action or clinical relevance for the sake of interpretability (in clinical trials or FDA approved; n=245) for correlation and elastic net analyses. The SMBPLSR analysis required further processing of the drug sensitivity data, which is described in the corresponding section. Dose-response experiments performed as part of this study were processed in the same way. In particular, departures from Loewe additivity[27] for combination treatments of MET and

MST1R (using Tepotinib and MK-2461, respectively) were quantified using the Combination Index (CI)[28] as implemented in the R package drc v3.0-1[29]:

$$CI = \frac{(D_C)_1}{(D_S)_1} + \frac{(D_C)_2}{(D_S)_2} \tag{4}$$

Here, $(D_C)_1$ and $(D_C)_2$ are the combination doses of drug 1 and 2 which result in a particular effect (e.g. 50% growth inhibition), while $(D_S)_1$ and $(D_S)_2$ are the single doses of drug 1 and 2 which result in the same effect. A CI of less than 1 indicates synergy, a CI equal to 1 indicates additivity and a CI of more than 1 indicates antagonism. Plotting the CI (and its confidence interval) versus different fractional effects (e.g. 20%, 40%, 60%, 80% and 100% growth inhibition) results in the so-called CI plot (see Supplementary Figure 4B). See the section on Cell viability assays for the exact concentrations of Tepotinib and MK-2461 used for single-compound and combination experiments.

Correlation network analysis

We used weighted gene correlation network analysis (WGCNA)[30] as implemented in the WGCNA R package v1.51 in order to find groups of proteins and/or p-sites, the intensity profiles of which were highly correlated across cells in the NCI60 or CRC65 cell line panels and might therefore be functionally related. WGCNA was carried out separately for each cell line panel. First, we merged the protein and p-site abundance matrices and z-transformed the resulting matrix in order to be also able to find proteins with high correlation to p-sites and *vice versa*. Next, we calculated signed co-expression similarities between all pairs of proteins/p-sites using the following formula:

$$s_{ij} = \frac{1 + cor(x_i, x_j)}{2} \tag{5}$$

Here, s_{ij} is the signed co-expression similarity between a protein/p-site x_i and a second protein/p-site x_j based on their Pearson correlation. Proteins/p-sites with high similarities show either a strongly positive or a strongly negative correlation across cell lines in the corresponding panel. We only considered similarities based on at least seven pairwise-complete observations from the resulting similarity matrix (symmetrical

along its diagonal). This ad-hoc cutoff was determined empirically by examining the distributions of similarities as a function of the number of pairwise-complete observations. Subsequently, we calculated several adjacency matrices $A = [s_{ij}]$ according to the following formula:

$$a_{ij} = s_{ij}^{\beta} \tag{6}$$

Here, a_{ij} is the adjacency between a protein/p-site x_i and a second protein/p-site x_j. The adjacency function parameter β was selected empirically by examining the scale-free topology of the resulting networks as a function of β as described previously[30]. We selected β to be 12 for the construction of signed co-expression networks for both the CRC65 and NCI60 cell line panel. Subsequently, we calculated the topological overlap matrix $\Omega = [\omega_{ij}]$ of these networks, which is a measure of how similar two nodes are with respect to their relative inter-connectedness:

$$\omega_{ij} = \frac{l_{ij} + a_{ij}}{min\{k_i, k_j\} + 1 - a_{ij}} \tag{7}$$

Here, contrary to unweighted networks, $l_{ij} = \sum_u a_{iu}a_{uj}$ instead of the number of nodes to which both *i* and *j* are connected and $k_i = \sum_u a_{iu}$ instead of the number of direct connections from *i* to other nodes. We then used the topological-overlap-based dissimilarity $D = [1 - \omega_{ij}]$ as an input for hierarchical clustering with average linkage. Subsequently, correlation clusters consisting of proteins/p-sites were detected by employing adaptive branch pruning using the Dynamic Hybrid method and requiring at least 30 members per module, whilst respecting the dendrogram topology during PAM operations[31]. Afterwards, similar clusters (correlation of their first principal components bigger than 0.9) were merged, followed by the calculation of the enrichment of functional annotations from Corum[32], GO[33], KEGG[34] and Reactome[35] in each cluster using Fisher's Exact Test. In order to associate both correlation clusters and functional categories with microsatellite instability (CRC65) or tissue of origin (NCI60), we aggregated protein/p-site intensities in these clusters/categories using gene set variation analysis as implemented in the GSVA R package v1.24.0[36]. Subsequently, we tested for differential expression of correlation clusters or functional categories in each cell line group (e.g. MSI+ or Lung) relative to the mean of all other groups using the R package limma v3.32.2[37]. We define full circles as groups of functionally and abundance-related

proteins(/p-sites), which are both significantly differentially abundant between groups of MSI- and MSI+ cells and also have a significant overlap in terms of their protein members. We also directly tested for differential abundance of proteins/p-sites belonging to specific correlation clusters in each cell line group relative to the mean of all other groups using limma (related to Figure 2F). All p-values were adjusted for multiple testing by calculating FDRs[38].

Calculation of Pathway and ATLANTiC scores

We used Pathway and ATLANTiC scores to rank pathways/kinases according to their importance in a given cell line. While Pathway scores reflect relative pathway activity, ATLANTiC scores integrate information on i) kinase abundance, ii) kinase phosphorylation and iii) kinase substrate phosphorylation to capture relative kinase activity. Below, we describe how Pathway scores were calculated and how the aforementioned sub-scores i-iii were computed and later aggregated to form ATLANTiC scores.

Pathway scores were calculated by first annotating p-sites on parent proteins with pathways and cancer hallmarks these proteins are involved in according to MsigDB[39]. Subsequently, we rolled-up p-site intensities to Pathway scores using z-score summation[40]. Briefly, we first standardized p-site expression profiles into z-scores over samples before aggregating them to form Pathway scores by summation and subsequent division by the square root of the number of proteins annotated with the respective pathway/hallmark. Finally, we filtered out pathways/hallmarks with less than six annotated p-sites.

For quantifying i) kinase abundance, we used MS1-based protein intensities from full proteome measurements after filtering out reverse hits as well as proteins, which were only identified by modification sites. Subsequently, we annotated each protein group with the gene name to which most of the detected peptides matched and log2-transformed the data. Next, we substituted missing values for each protein with half of the minimum intensity for this protein across the corresponding cell line panel (analogous to the LOD2 method[18]). Finally, we standardized the kinase abundance matrix into z-scores over samples.

For quantifying ii) kinase phosphorylation, we rolled-up MS1-based phosphopeptide intensities as described in the section Data post-processing and filtering. Subsequently, we annotated each protein group with the gene name to which most of the detected phosphopeptides matched and log2-transformed the data. Next, we substituted missing values for each phosphoprotein with half of the minimum intensity for this phosphoprotein across the corresponding cell line panel (analogous to the LOD2 method[18]). Finally, we standardized the phosphoprotein abundance matrix into z-scores over samples.

For quantifying iii) kinase substrate phosphorylation, we annotated p-sites on substrate proteins with the corresponding upstream kinase based on an integrated kinase substrate database (Supplementary Table 5) consisting of "KEA[41] [http://www.maayanlab.net/KEA2/gsl/kinase-substrate_phospho-site_level_set_library.tsv]" (downloaded on 2nd Aug 2016), "PhosphoELM[42] [http://phospho.elm.eu.org/dumps/phosphoELM_all_latest.dump.tgz]" (downloaded on 28th Apr 2015), "PhosphoNetworks[43] [http://www.phosphonetworks.org/download/highResolutionNetwork.csv]" (downloaded on 2nd Aug 2016), "PhosphoSitePlus[44] [https://www.phosphosite.org/downloads/Kinase_Substrate_Dataset.gz]" (downloaded on 13th Apr 2016), "SIGNOR[45] [https://signor.uniroma2.it/downloads.php]" (downloaded all data on 3rd Aug 2016) and "Uniprot[46] [ftp://ftp.uniprot.org/pub/databases/uniprot/current_release/knowledgebase/taxonomic_divisions/uniprot_sprot_human.dat.gz]" (downloaded on 2nd Aug 2016; see also Supplementary Table 5). Kinases with less than six annotated substrates in the individual kinase substrate databases above and kinases not present in the "Uniprot kinase list [https://www.uniprot.org/docs/pkinfam.txt]" (downloaded on 16th Aug 2013) were excluded from the integrated kinase substrate database. Matching of p-sites to kinases was performed at the level of substrate proteins after mapping database-specific kinase accessions to gene names using a combination of Uniprot and manual curation. Subsequently, we rolled-up p-site intensities to kinase-substrate-phosphorylation-scores using z-score summation as described above. Each of these scores was annotated with the corresponding kinase. Finally, we filtered out scores with less than six annotated p-sites.

All of these sub-scores i-iii were filtered for kinases using a table provided by "Uniprot[46] [https://www.uniprot.org/docs/pkinfam]" (downloaded on 14th Aug 2013), followed by restriction to kinases present in all three sub-scores and rescaling to [0,1].

In order to calculate ATLANTiC scores from the three sub-scores i-iii mentioned above, we calculated the geometric mean between them for each kinase in each cell line, followed by rescaling to [0,1].

The resulting matrices of the NCI60 and CRC65 cell line panels for pathways, sub-scores i-iii and our ATLANTiC scores can be explored at http://atlantic.proteomics.wzw.tum.de/QueryLandscape.html. For visual clarity, we smoothed these heatmaps using a kernel smoother for irregular 2-d data from the R package fields v9.6. Users can draw rectangles around regions of interest and are presented with a table quantifying the relative activity in the selected area. We define relative activity based on the non-smoothed data in relation to the corresponding maximum in the selected area. Here, we only consider data points the smoothed intensity of which exceeds a user-defined quantile threshold (Sea level).

Recapitulated biological pathways

In order to find out which biological pathways are better recapitulated at the proteome or phosphoproteome level, we focused on functional associations between any two proteins recorded in pathway databases and compared the correlation of these two proteins across the NCI60 and CRC65 cell line panel at the protein and phosphoprotein level. Pathway databases were extracted from the R package ESEA v1.0[47], which included KEGG[48], Biocarta, Reactome[35], NCI PID[49] and SPIKE[50], HumanCyc[51] and Panther[52]. We defined a functional association between two proteins from these databases to be "recapitulated" if the correlation coefficient across cell lines of the NCI60 or CRC65 panel was greater than 0.5 at the protein and/or phosphoprotein level. Since correlation coefficients calculated based only on a few cell lines tend to have higher variance, we restricted our analysis to correlations, which are based on at least seven cell lines. Finally, we tested for enrichment of functional associations in the set of edges recapitulated exclusively at the protein/phosphoprotein level or recapitulated at both levels using Fisher's Exact Test.

Outlier analysis

We performed outlier analysis in order to find (phospho)proteins/p-sites with substantially higher abundance in a particular cell line compared to all other cell lines. It is well known that transcriptional outliers can be potential therapeutic targets[24] and we postulate that such an analysis should be even more conclusive at the protein or p-site level. We classified (phospho)proteins/p-sites as outliers if one of the following criteria was met:

1. The p-site in question is a phosphorylated tyrosine
2. The maximal abundance of a particular (phospho)protein/p-site is at least five times higher in one cell line compared to the second highest abundance in all other cell lines of the same cell line panel
3. A particular (phospho)protein/p-site is only identified in a single cell line and its abundance is higher than a specific threshold

In order to determine this threshold, we first calculated empirical p-values quantifying the likelihood of measuring a (phospho)protein/p-site exactly once given its maximal abundance in one of our cell line panels. This is motivated by the fact that the number of missing values across cell lines and the maximal intensity of a (phospho)protein/p-site are negatively correlated. Briefly, for a (phospho)protein/p-site with a maximal abundance of i, all (phospho)proteins/p-sites with a maximal abundance in the range of $[i-\theta, i+\theta]$ were grouped into a set φ. The constant θ was used to control the number of (phospho)proteins/p-sites in φ. In our analysis, θ was set to 0.05 for protein and p-site data and to 0.2 for phosphoprotein data. The empirical p-value p_{si} for a single maximal abundance is then calculated as $p_{si} = \frac{n^1}{n}$, where n^1 is the number of proteins measured only once across all cell lines in set φ and n is the total number of (phospho)proteins/p-sites in set φ. Next, we trained LOcally WEighted Scatterplot Smoothing (LOWESS) models[53] using i, the maximal abundance of (phospho)proteins/p-sites, as the independent variable and p_{si} as the dependent variable. In general, the number of missing values and the maximal abundance of (phospho)proteins/p-sites are anti-correlated, resulting in monotonically decreasing fitted

models. Finally, we used these LOWESS models to predict the maximal abundance corresponding to $p_{si} = 0.1$, which we subsequently used as the aforementioned threshold. In the CRC65 dataset, these thresholds were 7.17 (quantile = 42%), 23.31 (quantile = 21%) and 5.52 (quantile = 23%) for p-sites, phosphoproteins and proteins, respectively. In the NCI60 dataset, the corresponding thresholds were 7.30 (quantile = 50%), 24.40 (quantile = 30%) and 5.96 (quantile = 21%).

A compound in the DTP dataset was selected as an outlier if the GI50 value of the most sensitive cell line was 1000 times lower compared to all other cell lines of the same cell line panel.

Simple correlation and elastic net analyses

We applied 1) simple correlation analysis and 2) elastic net regression[54] to discover proteins and p-sites predicting drug response:

1) For any given drug, we calculated correlation coefficients between the corresponding drug response data and the abundance data for all proteins/p-sites across the respective cell line panel. Here, we excluded pair-wise incomplete observations and refrained from imputing missing values in both drug response and protein/p-site data. In addition, we only considered drug-protein/p-site pairs with more than seven pair-wise complete observations in order to avoid artificially high/low correlations due to low sample size. All p-values displayed in our ATLANTiC web portal were adjusted for multiple testing by calculating FDRs[38].

2) To identify linear combinations of multiple proteins/p-sites predicting drug response, we applied elastic net regression as described earlier[2]. Briefly, elastic net regression combines L1 and L2 penalties in order to alleviate problems commonly encountered while modelling (prote)omics data, such as large numbers of predictors p (proteins/p-sites) and small numbers of samples n (p>>n). On the one hand, the L2 penalty shrinks most of the regression coefficient towards zero, but rarely to exactly zero. In contrast, the L1 penalty reduces most regression coefficients to exactly zero and only keeps a few predictors with non-zero coefficients, which improves the biological interpretability of the final model. Elastic net regression takes

advantage of both penalties and therefore forces most of the correlation coefficients to zero and – at the same time – selects only a subset of predictors showing high correlation to the dependent variable (drug response). Double shrinkage of coefficients is prevented by the introduction of a scaling factor[54] while the hyperparameter $\alpha \in [0, 1]$ is used to control the balance between the L2-penalty (α=0) and the L1-penalty (α=1). In addition, a second hyperparameter λ controls the degree of regularization[55]. In our analysis, α was set to 0.05 and λ was optimized using leave-one-out cross-validation with mean-squared-error as the loss function. We used a bootstrapping approach to select robust (phospho)proteomic biomarkers predicting drug sensitivity or resistance. To do so, 100 bootstrap samples of cell lines were generated for each drug, followed by model fitting as described above. Subsequently, the results were summarized using two statistics: the regression coefficient referred to as "effect size" and the selection frequency, which is essentially the number of times a protein/p-site was part of the fitted models. Since these models were trained independently on each drug, we z-transformed the effect sizes in order to facilitate the comparison of effect sizes between different models. Elastic net regression models were fit using the R package glmnet v2.0-16[55]. We used drug sensitivity information for both cell line panels and imputed missing values as described earlier. Drugs for which we had less than 12 response measurements in a cell line panel were excluded in order to avoid the selection of non-robust biomarkers.

For the evaluation of general markers (related to Figure 4A), we calculated weighted sum scores. We defined the primary score for a sensitivity marker as $s^+ = f^+ \cdot n^+$ and the primary score for a resistance marker as $s^- = f^- \cdot n^-$, where f is the effect size and n is the selection frequency in elastic net regression models of a particular drug sensitivity dataset. The weight for primary scores is defined as $w = \left| log2\left(\frac{|s^+|+\alpha}{|s^-|+\alpha}\right)\right|$. Here, an offset $\alpha = 100$ was added to the log-ratio in order to avoid extreme values resulting from very small denominators. The weighted sum scores for sensitivity and resistance markers were defined as $s^+ \cdot w$ and $s^- \cdot w$, respectively.

Network analysis using STRING

Proteins associated with resistance to Arsenic trioxide that were included in more than 50 bootstrap models were selected and uploaded to the STRING database[56]. The corresponding sub-network is shown in Figure 4B.

Random forest models predicting 5FU response

We downloaded drug response data for 77 CRC cell lines from the supplement of a study focusing on 5-fluorouracil (5FU)[25], which is a commonly used chemotherapeutic in colorectal cancer. Subsequently, we modeled the response to 5FU of 60 of these cell lines (the intersection with the CRC65 cell line panel) as a function of the abundance of proteins involved the metabolism of 5FU: TYMP, TK1, UMPS, PPAT, UPP1, UCK2, UCK1, RRM1, RRM2, TYMS and DPYD, as well as three drug transporters SLC29A1, ABCC3, ABCC4.

We used the R package randomForest v4.6-12[57] to fit random forest models using combinations of at least two and up to 14 (all) proteins to the data. For each model, 10 trees were grown (ntree=10) and leave-one-out cross-validation was used to evaluate the prediction accuracy as measured by the sum of squared errors, i.e. cross-validation error. We observed that, comparing to signatures combining two proteins, the cross-validation error decreased dramatically when three proteins are included and stopped decreasing when more than seven proteins are included. Therefore, we selected the top five models from all combinations of three to seven proteins and aped them to predict the sensitivity towards 5FU for all NCI60 cell lines.

Analysis of similar drug response profiles

We extended sparse multi-block partial least squares regression (SMBPLSR)[58] in order to take advantage of the correlation structure of the drug sensitivity datasets we integrated with our (phospho)proteomics data. Sparse partial least square regression (SPLSR) can be used to select a set of independent variables

predicting multiple (correlated) dependent variables. However, our phosphoproteomics data is biased by our proteomics data in the sense that p-sites often correlate well with the corresponding protein abundance, which may in turn bias SPLSR models towards selecting multiple p-sites from the same protein. To solve this problem, we rolled-up phosphopeptide abundances to phosphoprotein abundances (see the "Data processing and filtering" part for more details). Next, we correlated the phosphoprotein intensity to the intensities of all matching p-sites across both cell line panels. In cases where this correlation was low (Pearson's R<0.5), we labelled the respective p-site as divergent. Both phosphoprotein intensities and intensities of divergent p-sites were used in this analysis. This resulted in two input matrices, one for phosphoproteins and one for divergent p-sites. Multi-block Partial Least Squares regression (MBPLSR) can now be used to perform predictive modelling of one dependent dataset (drug response) as a function of multiple independent datasets (phosphoproteins and divergent p-sites). MBPLSR is a generalization of partial least squares regression (PLSR) and aims at linking multiple independent datasets with one dependent dataset as opposed to considering only a single independent dataset. A recent extension of MBPLSR called sparse MBPLSR (SMBPLSR) added regularization for feature selection[58], which improves the interpretability of fitted models. Here, we briefly introduce SMBPLSR, and subsequently present an additional extension of it.

As an input, SMBPLSR requires a set of omics datasets $\mathbf{X^1}, \dots, \mathbf{X^b}, \dots, \mathbf{X^B}$ with rows corresponding to samples (cell lines) and columns corresponding to measured biomolecules (proteins and divergent p-sites) and a phenotypic dataset $\mathbf{Y}$ with rows corresponding to the same samples in $\boldsymbol{X}$s and columns corresponding to (quantitative) phenotypic variables (drug responses). We aim at using divergent p-sites and phosphoproteins (variables in $\mathbf{X}$s) to predict drug responses (variables in $\mathbf{Y}$), thereby associating a small number of candidate p-sites and/or phosphoproteins with multiple drugs having correlated responses across our cell line panels. SMBPLSR solves this problem by optimizing the following loss function:

$$M = \min_{w,c} \left| \left| \mathbf{M} - \mathbf{wc}' \right| \right|_F^2 + P_\lambda(\mathbf{W}) \quad (8)$$

Here, $\mathbf{M} = \mathbf{X}'\ \mathbf{Y}$ while $\mathbf{X}$ results from concatenating all $\mathbf{X}$s, i.e. $\mathbf{X} = [\mathbf{X}^{\mathbf{1}}, \dots, \mathbf{X}^{\boldsymbol{b}}, \dots, \mathbf{X}^{\boldsymbol{B}}]$. In addition, $\mathbf{w}$ and $\boldsymbol{c}$ denote the loading weights (similar to regression coefficients in ordinary least squares regression) of $\mathbf{X}$ and $\mathbf{Y}$, respectively. We denote the matrix consisting of $\mathbf{w}$ and $\mathbf{c}$ as $\mathbf{W}$ and $\mathbf{C}$ (see below). It is worth mentioning that $||..||_F$ is the Frobenius norm and $P_\lambda(\mathbf{W})$ is the penalty factor introducing sparsity in $\mathbf{w}$ (regularization). As a result, most loading weights of variables in $\mathbf{X}$ are 0, meaning that they do not influence the final model. Variables with non-zero loading weights are selected and contribute to the final model.

We extended SMBPLSR with a second penalty $P_\theta(\mathbf{C})$, which also leads to sparsity in $\boldsymbol{c}$ **(**drug response matrix**)**. Since we do not assume that a small number of p-sites and/or phosphoproteins is able to predict the response of cell lines to *all* drugs, we aimed at only selecting the drugs showing the highest correlation to variables in $\mathbf{X}$s. We hypothesized that drugs selected this way might have similar modes of action. This resulted in the extended loss function below:

$$M = \min_{w,c} \left|\left|\mathbf{M} - \mathbf{w}\mathbf{c}'\ \right|\right|_F^2 + P_\lambda(\mathbf{W}) + P_\theta(\mathbf{C}) \tag{9}$$

To implement this new loss function, we modified the algorithm in table 2 from an earlier publication[58] as follows:

Original:

$$(viii)\ \ \mathbf{c}_a = \mathbf{Y}'_{a-1}\mathbf{t}_a/||\mathbf{T}'_a\mathbf{u}_{\boldsymbol{a}}|| \tag{10}$$

Our modification:

$$(viii)\ \ \mathbf{c}_a = \mathrm{ST}(\mathbf{Y}'_{a-1}\mathbf{t}_a/||\mathbf{T}'_a\mathbf{u}_{\boldsymbol{a}}||) \tag{11}$$

Here, ST is the soft-thresholding operator.

We only focused on solid tumors in this analysis since the phosphoproteomic and drug response data of leukemia cell lines were very different from the rest of the cell lines in the NCI60 panel. Otherwise, most of the loading weights (components) would be purely driven by these cell lines. In addition, we excluded drugs to which all the remaining cell lines showed substantial resistance ($-\log_{10}$(GI50) < 1.5), which retained

172 drugs. It is worth noting that we z-score transformed all input matrices (drug responses, phosphoproteins and p-sites) prior to model fitting. We used 5-fold cross validation to optimize the number retained variables from our input matrices. Possible numbers of variables to keep were 2, 4, 6, 8, 10, 12, 14, 16, 18 and 20 for drugs and 50, 100, 200 and 400 for phosphoproteins and p-sites, respectively.

Survival analysis

In order to determine the effect of AK1 on patient survival, we stratified the AML cohort into AK1 positive and negative patients. Overall survival (OS) of these groups was estimated using the Kaplan–Meier method, followed by a comparison of the two curves using a two-sided log-rank test. We used Cox proportional hazards models to evaluate the association of AK1 and other potential confounding clinical factors with OS. For the breast cancer cohort, we used H-scores to stratify patients into groups based on their pPGR status. Overall survival was estimated as described above for AK1. We optimized the H-score threshold above which patients were considered pPGR positive to minimize the p-value of the two-sided log-rank test comparing the survival of the two groups. As described for AK1, we used Cox proportional hazards models to evaluate the association of PGR/pPGR and other potential cofounding factors with OS.

Statistics

All statistical tests performed in this study were two-sided if applicable. In box plots, the lower and upper boundary of boxes indicate the 25% and 75% quantile, respectively. Whiskers represent 1.5x the interquartile range and any data point outside of this range is considered an outlier and plotted individually.

SUPPLEMENTARY FIGURES

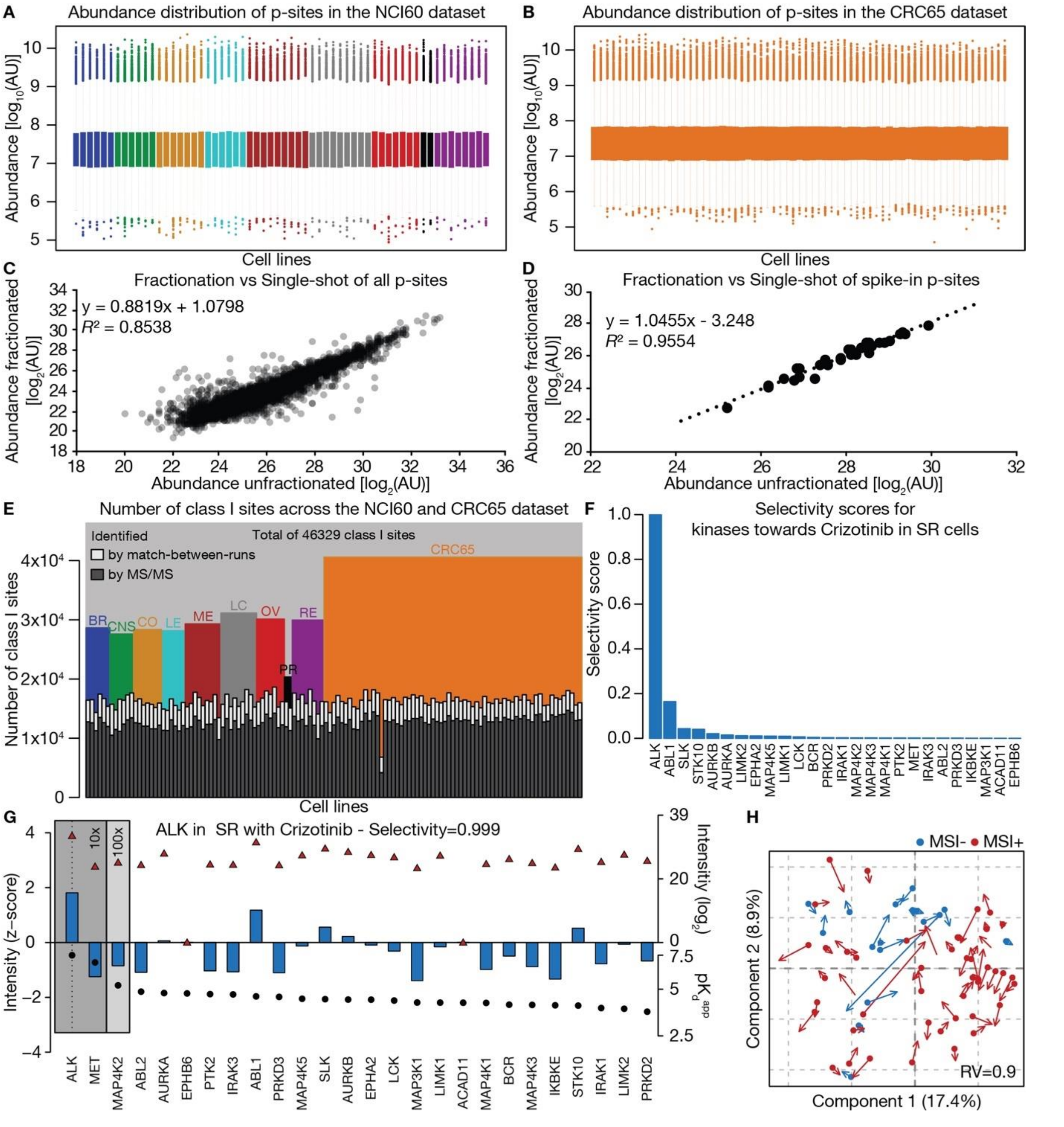

Supplementary Figure 1. Exploratory analysis of (phospho)proteomes of 125 cancer cell lines. (**A**) & (**B**) Boxplots showing the distribution of log-transformed p-site abundance in the (**A**) NCI60 (n=60 cell lines) and (**B**) CRC65 dataset (n=65 cell lines). Cell lines are colored by tissue of origin as in Figure 1. (**C**) & (**D**) Comparison of p-site abundance from measurements of a fractionated sample and a single-shot

measurement without fractionation of HeLa cells. The overall correlation is high for (**C**) all p-sites (n=4,935 p-sites) and (**D**) spike-in p-sites (n=34 p-sites), suggesting that fractionation of phosphopeptides does not interfere with the quantification of p-sites. (**E**) Bar plot visualizing the number of quantified class-I p-sites (at least 75% localization probability, Supplementary Methods) across both cell line panels (n=125 cell lines). Bars are split into p-sites identified by match-between-runs or MS/MS. Combined bars visualize the sum of p-sites within each tumor entity and are colored by tissue of origin as in Figure 1. (**F**) Example use case for the online ATLANTiC web application. The (phospho)proteome landscapes of cell lines generated in this work can be combined with phenotypic drug effects on cell lines and the previously published target landscape of kinase inhibitors to identify the target protein responsible for the observed phenotype. For example, SR cells are very sensitive to Crizotinib. To identify the most likely protein target responsible for the phenotype, the targets of Crizotinib are ordered by decreasing selectivity score (Supplementary Methods). ALK has a selectivity score of 0.999 towards Crizotinib in SR cells. (**G**) The selectivity plot enables further evaluation of the selectivity score. In this plot, the targets of Crizotinib are ordered by binding strength (expressed by their pK_d^{app}; $-\log_{10}$ of the apparent K_d values; black dots; right bottom y-axis). The shaded areas enclose kinases whose pK_d^{app} values are within 10-fold (dark gray) or 100-fold (light gray) of the pK_d^{app} of the most potent Crizotinib target ALK (Supplementary Methods for additional details). The left y-axis shows the z-scored intensities of kinases targeted by Crizotinib across the NCI60 panel as bars and their log2-transformed intensities as red triangles (right top y-axis). It is apparent that ALK is relatively more abundant than the next best Crizotinib targets MET and MAP4K2. In fact, ALK accounts for 99.9% of the proteomic abundance of the top three Crizotinib targets (expressed as a selectivity score), making ALK inhibition by far the most likely molecular target explaining the sensitivity of SR cells towards Crizotinib. A related analysis provided by the ATLANTiC web application is to select a protein of interest first, and the output is a set of kinase inhibitors and cell lines along with the same selectivity score, which can guide the selection of a suitable cell line to study the drug/protein interaction in more detail. (**H**) Multiple co-inertia analysis of the phospho- and full proteome data of the CRC65 panel (blue=MSI- or microsatellite instability negative, n=17, red=MSI+ or microsatellite instability positive, n=47 cell lines).

The first two components are shown. Bases of arrows represent the full proteome and ends of arrows represent the phosphoproteome of a given cell line. Short arrows indicate a good correlation between phospho- and full proteomes. Related to Figure 1A and 1D. Source data are provided as a Source Data file.

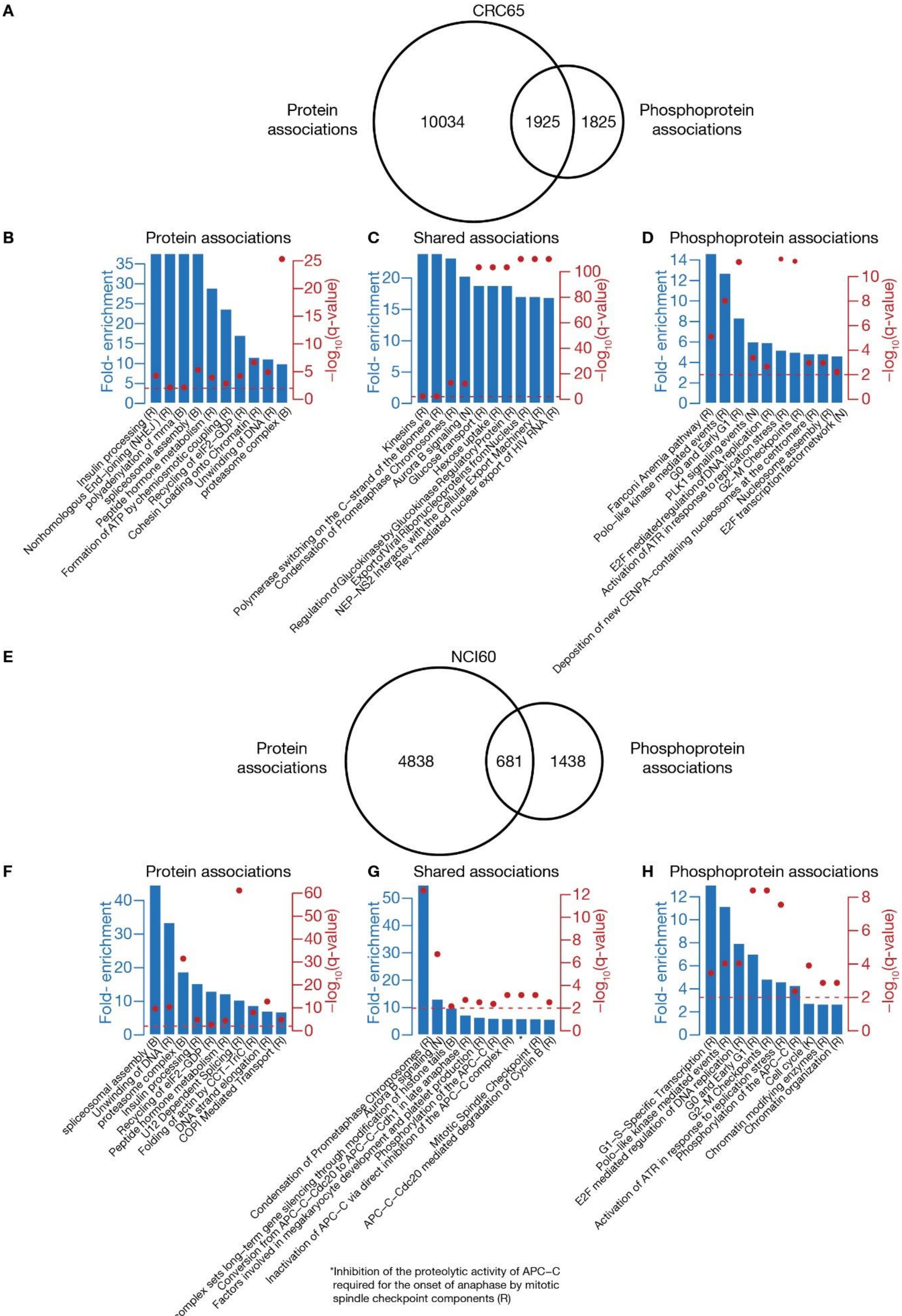
A
CRC65
Protein associations
10034
1925
1825
Phosphoprotein associations
B
Protein associations
Fold- enrichment
−log10(q-value)
Insulin processing (R)
Nonhomologous End-joining (NHEJ) (R)
polyadenylation of mrna (B)
spliceosomal assembly (B)
Peptide hormone metabolism (R)
Formation of ATP by chemiosmotic coupling (R)
Recycling of eIF2-GDP (R)
Cohesin Loading onto Chromatin (R)
Unwinding of DNA (R)
proteasome complex (B)
C
Shared associations
Kinesins (R)
Polymerase switching on the C−strand of the telomere (R)
Condensation of Prometaphase Chromosomes (R)
Aurora B signaling (N)
Glucose transport (R)
Hexose uptake (R)
Regulation of Glucokinase by Glucokinase Regulatory Protein (R)
Export of Viral Ribonucleoproteins from Nucleus (R)
NEP−NS2 Interacts with the Cellular Export Machinery (R)
Rev−mediated nuclear export of HIV RNA (R)
D
Phosphoprotein associations
Fanconi Anemia pathway (R)
Polo−like kinase mediated events (R)
G0 and Early G1 (R)
PLK1 signaling events (N)
E2F mediated regulation of DNA replication (R)
Activation of ATR in response to replication stress (R)
G2−M Checkpoints (R)
Deposition of new CENPA−containing nucleosomes at the centromere (R)
Nucleosome assembly (R)
E2F transcription factor network (N)
E
NCI60
Protein associations
4838
681
1438
Phosphoprotein associations
F
Protein associations
spliceosomal assembly (B)
Unwinding of DNA (R)
proteasome complex (B)
Insulin processing (R)
Recycling of eIF2−GDP (R)
Peptide hormone metabolism (R)
U12 Dependent Splicing (R)
Folding of actin by CCT−TriC (R)
DNA strand elongation (R)
COPI Mediated Transport (R)
G
Shared associations
Condensation of Prometaphase Chromosomes (R)
Aurora B signaling (N)
The prc2 complex sets long−term gene silencing through modification of histone tails (B)
Conversion from APC−C−Cdc20 to APC−C−Cdh1 in late anaphase (R)
Factors involved in megakaryocyte development and platelet production (R)
Phosphorylation of the APC−C (R)
Inactivation of APC−C via direct inhibition of the APC−C complex (R)
*
Mitotic Spindle Checkpoint (R)
APC−C−Cdc20 mediated degradation of Cyclin B (R)
H
Phosphoprotein associations
G1−S−Specific Transcription (R)
Polo−like kinase mediated events (R)
E2F mediated regulation of DNA replication (R)
G0 and Early G1 (R)
G2−M Checkpoints (R)
Activation of ATR in response to replication stress (R)
Phosphorylation of the APC−C (R)
Cell cycle (K)
Chromatin modifying enzymes (R)
Chromatin organization (R)
*Inhibition of the proteolytic activity of APC−C required for the onset of anaphase by mitotic spindle checkpoint components (R)

Supplementary Figure 2. Related to Figure 1E – Baseline (phospho)proteomes recapitulate distinct biological pathways. (**A**) Venn diagram showing shared and unique interactions recapitulated at the protein and phosphoprotein level in the CRC65 cell line panel. We defined a functional association between two proteins to be recapitulated if their intensities were correlated at either the protein or phosphoprotein level (Pearson correlation coefficient > 0.5 across at least seven cell lines; Supplementary Methods). (**B**-**D**) Biological pathways significantly (Fisher's exact test; Benjamini-Hochberg corrected P < 0.05) enriched in interactions (**B**) exclusive to the protein level, (**C**) detected at both levels and (**D**) exclusive to the phosphoprotein level (R – Reactome; K – KEGG; N – NCI; B – Biocarta). (**E**-**H**) The same as in panels A-D but for the NCI60 panel. Representative pathways are shown in Figure 1E. Source data are provided as a Source Data file.

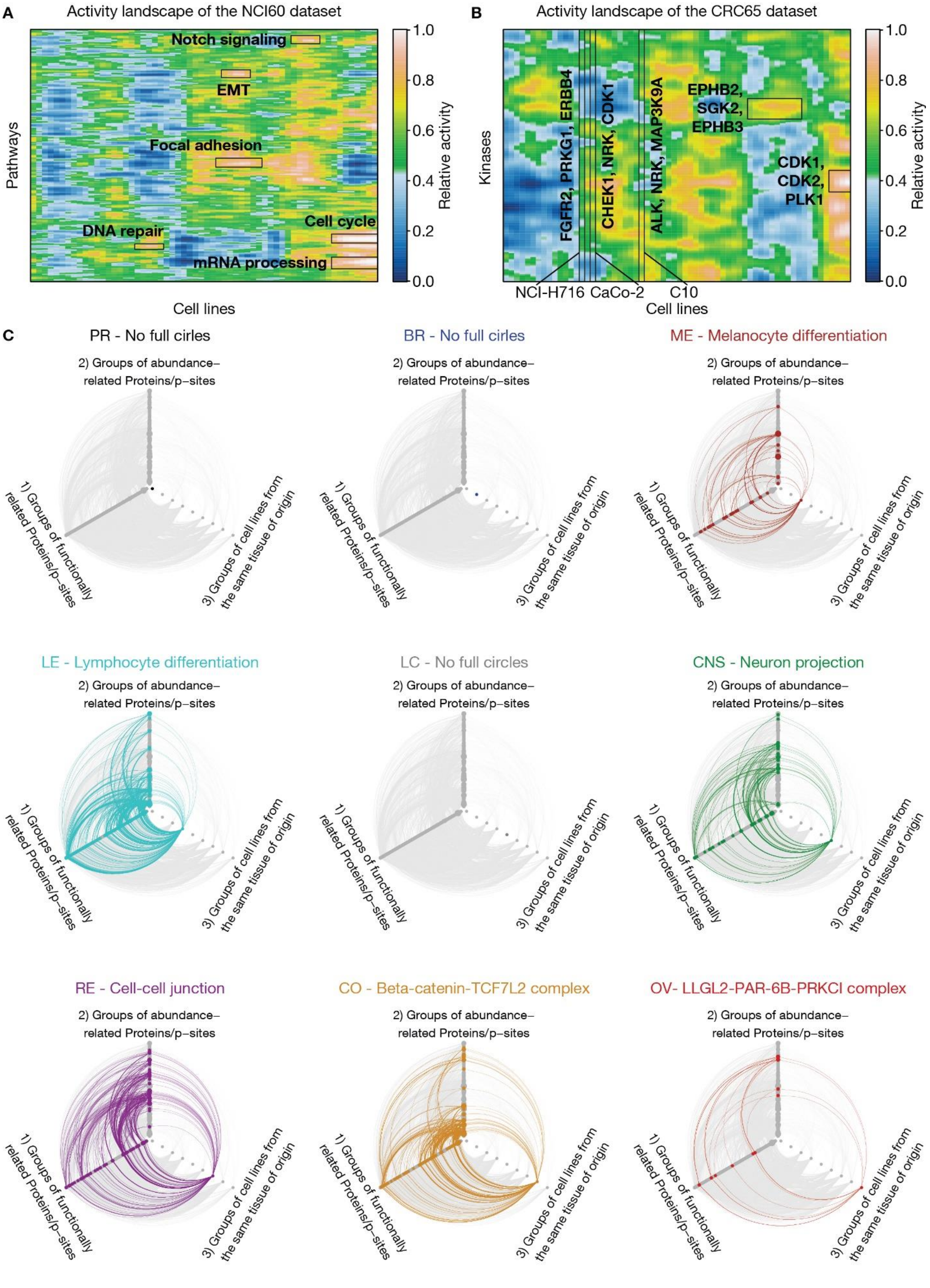
A
Activity landscape of the NCI60 dataset
Notch signaling
EMT
Focal adhesion
DNA repair
Cell cycle
mRNA processing
Pathways
Cell lines
Relative activity
1.0
0.8
0.6
0.4
0.2
0.0
B
Activity landscape of the CRC65 dataset
FGFR2, PRKG1, ERBB4
CHEK1, NRK, CDK1
ALK, NRK, MAP3K9A
EPHB2, SGK2, EPHB3
CDK1, CDK2, PLK1
Kinases
NCI-H716
CaCo-2
C10
Cell lines
Relative activity
C
PR - No full cirles
BR - No full cirles
ME - Melanocyte differentiation
LE - Lymphocyte differentiation
LC - No full circles
CNS - Neuron projection
RE - Cell-cell junction
CO - Beta-catenin-TCF7L2 complex
OV- LLGL2-PAR-6B-PRKCI complex
1) Groups of functionally related Proteins/p−sites
2) Groups of abundance−related Proteins/p−sites
3) Groups of cell lines from the same tissue of origin

Supplementary Figure 3. Related to Figure 2 – Activity landscapes and correlation networks. (**A**) Activity landscape of cellular pathways for the NCI60 panel (Supplementary Methods; n=60 cell lines) and (**B**) of kinases for the CRC65 panel (n=64 cell lines). Relative activity ranges from 0 to 1 representing minimal and maximal relative activity, respectively. Areas of high activity are highlighted (Supplementary Methods). (**C**) Hive plot showing significant associations between groups of functionally related proteins/p-sites, cell lines from the same tissue of origin and groups of abundance-related proteins/p-sites (highly correlated proteins and p-sites) identified by weighted gene correlation network analysis in the NCI60 dataset (Supplementary Methods). Colored edges (colored according to tissue of origin as in Figure 1) represent significant associations between corresponding nodes on all three axes. The title of each sub-panel indicates the tissue of origin and includes a representative annotation. There was no full circle in highly heterogeneous tumor types (e.g. breast cancer and lung cancer), indicating that groups of functionally related proteins/p-sites enriched in groups of abundance-related proteins/p-sites cannot be explained by the tissue of origin in these cases. However, groups of abundance-related proteins/p-sites highly abundant in more homogeneous tumor subtypes (e.g. leukemia and melanoma) are often dominated by proteins/p-sites with functions related to their tissues of origin. For example, the functional annotation Melanocyte differentiation is enriched in a group of abundance-related proteins/p-sites, which are themselves highly abundant in melanoma cell lines. These results suggest that highly correlated proteins/p-sites reflect the tissue-specific biology of tumor cell lines derived from homogenous tissues. However, they do not reflect the tissue-specific biology of heterogeneous tissues (see also Figure 1D). Source data are provided as a Source Data file.

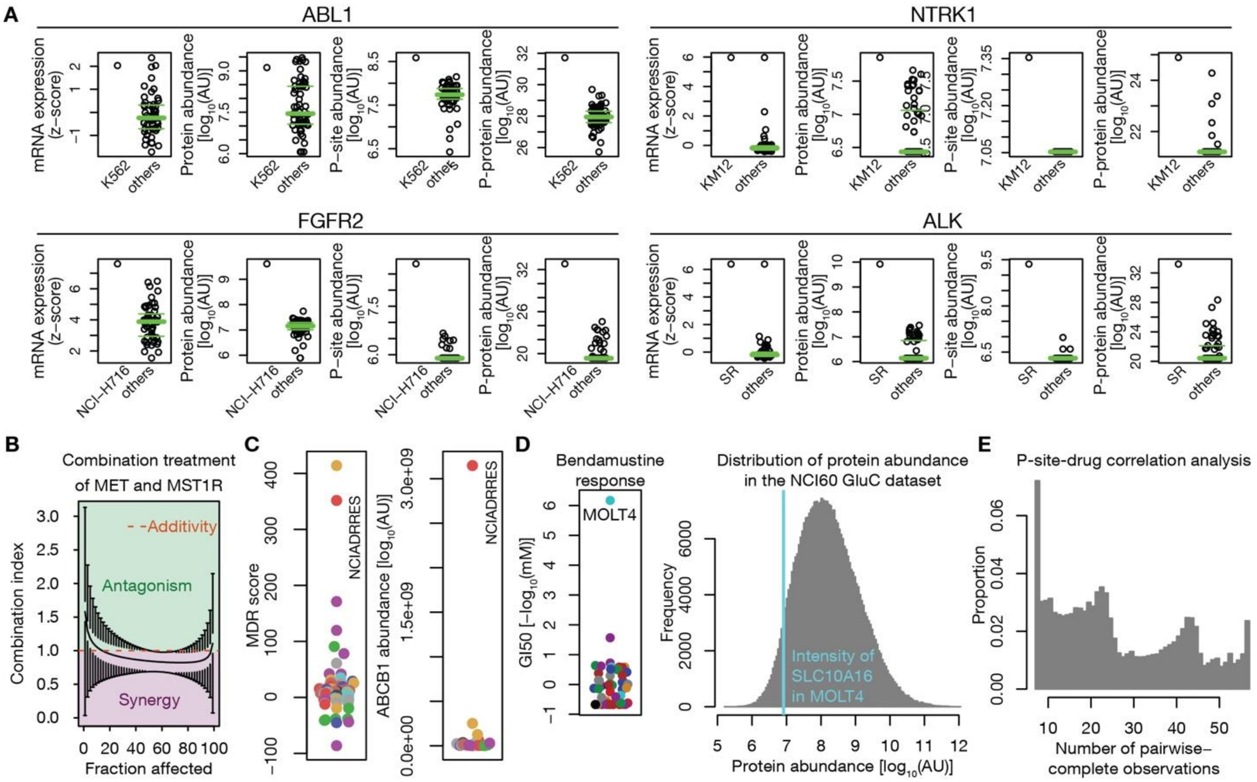

Supplementary Figure 4 – Outlier proteins and p-sites and their indications for drug responses. (**A**) Beeswarm plots highlighting outlier cell lines expressing high levels of selected kinases (ABL1, NTRK1, FGFR2 and ALK). The abundance of the gene products is compared at the mRNA (the average z-score of 5 platforms in CELLMINER[20]), protein, p-site and phosphoprotein levels (ABL1 mRNA n= 61; Protein, P-site, P-protein n=60 cell lines; NTRK1 mRNA n=61; Protein, P-site, P-protein n=60 cell lines; FGFR2 mRNA n=57; Protein, P-site, P-protein n=65 cell lines; ALK mRNA: n= 61; Protein, P-site, P-protein n=60 cell lines. (**B**) Combination index (CI) plot visualizing the CI as a function of the Fraction affected for the combination treatment of HDC-8 cells with Tepotinib (targeting MET) and MK-2461 (targeting MST1R; Supplementary Methods). Error bars represent the 95% confidence interval of the CI. The area shaded in purple indicates synergistic effects of the two drugs, the orange dotted line indicates additive effects and the green area indicates antagonistic drug effects. (**C)** The multi-drug resistance score (MDR) of NCI60 cell lines as measured by the "NCI [https://discover.nci.nih.gov/cellminer/celllineMetadata.do]". NCIADRRES is one of the two high MDR score cell lines (left panel; n=58 cell lines), which can potentially be explained by very high expression of the multi-drug exporter ABCB1 (right panel; n=58 cell lines). Cell

lines are colored by tissue of origin as in Figure 1. (**D**) MOLT4 is an outlier cell line when considering response to Bendamustine in the NCI60 panel (left panel; n=60 cell lines; GI50=growth inhibitory concentration analogous to an IC50). Cell lines are colored by tissue of origin as in Figure 1. The right panel shows a histogram visualizing the distribution of intensities from our Glu-C digests in the NCI60 panel. The abundance of SLC10A16, which was exclusively identified in MOLT4, is indicated by a light blue line and might explain MOLT4's response to Bendamustine. (**E**) Histogram showing the distribution of the number of pairwise-complete observations based on which correlations for p-site-drug combinations were calculated (n=3,417,931 p-site-drug combinations). Source data are provided as a Source Data file.

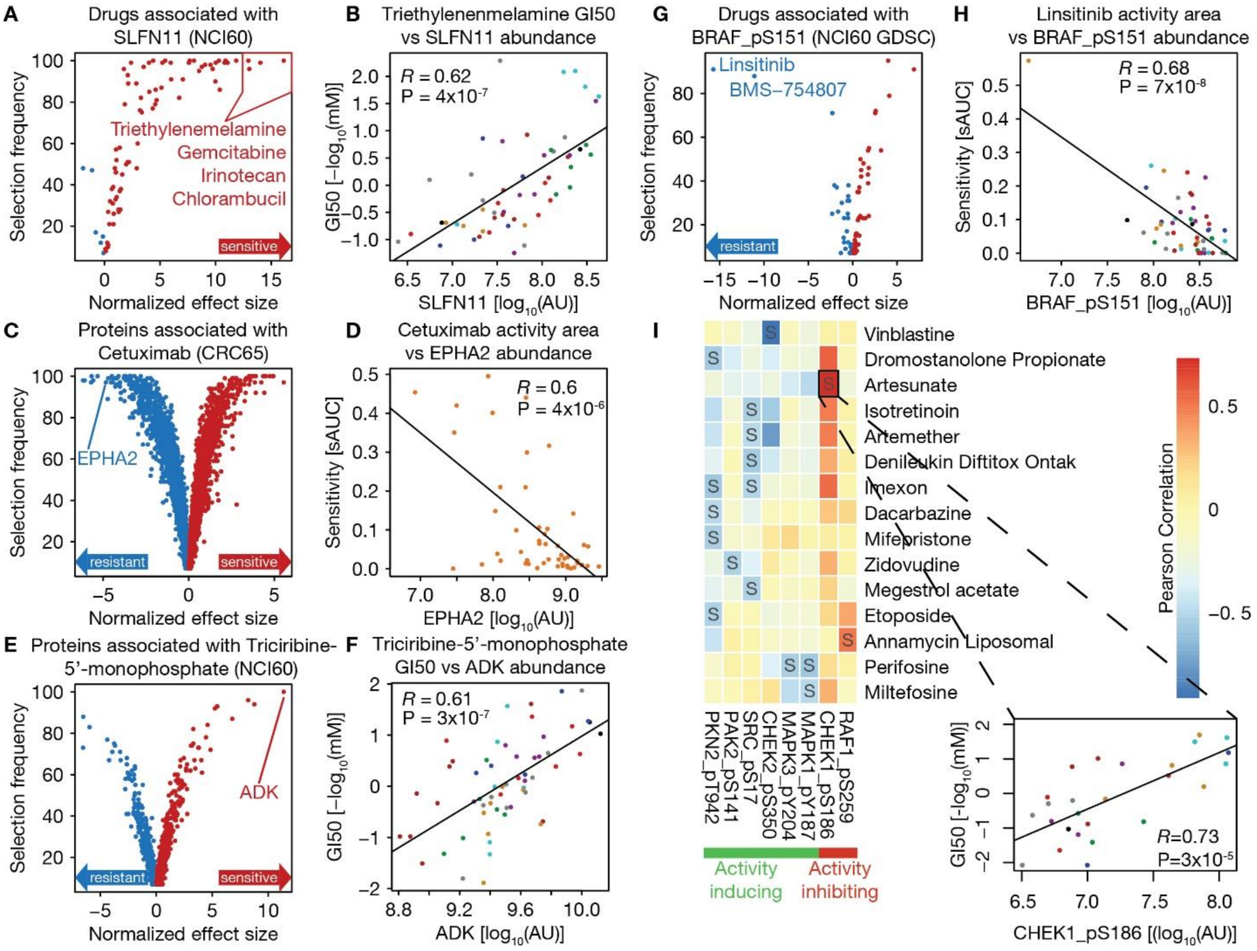

Supplementary Figure 5 – Correlation-based markers and their indication for drug sensitivity. (**A**) Volcano plot showing that SLFN11 is a sensitivity marker (red) for multiple inhibitors targeting DNA, such as Triethylenemelamine, Gemcitabine, Irinotecan and Chlorambucil. (**B**) SLFN11 abundance in the NCI60 panel is significantly (Pearson correlation test; $P < 0.05$) positively correlated with Triethylenemelamine sensitivity (right panel; n=59 cell lines; GI50=growth inhibitory concentration analogous to an IC50). Cell lines are colored by tissue of origin as in Figure 1. (**C**) Volcano plot highlighting that EHPA2 is a resistance marker (blue) for Cetuximab (left panel). (**D**) EPHA2 abundance in the NCI60 panel is significantly (Pearson correlation test; $P < 0.05$) negatively correlated with Cetuximab sensitivity (right panel; n=65 cell lines; sAUC=1-standardized area under the dose-response curve). Cell lines are colored by tissue of origin as in Figure 1. (**E**) Volcano plot showing that ADK is the top selected sensitivity marker for Triciribine-5'-monophosphate (left panel). (**F**) ADK protein abundance in the NCI60 panel is significantly

(Pearson correlation test; $P < 0.05$) positively correlated with Triciribine-5'-monophosphate sensitivity (right panel; n=59 cell lines). Cell lines are colored by tissue of origin as in Figure 1. (**G**) Volcano plot depicting drugs associated with BRAF_pS151. These include Linsitinib and BMS-754807. (**H**) Linsitinib is significantly (Pearson correlation test; $P < 0.05$) negatively correlated with BRAF_pS151 intensity (right panel; n=60 cell lines). Cell lines are colored by tissue of origin as in Figure 1. (**I**) Correlating the abundance of activity-regulating kinase p-sites with drug sensitivity can suggest potential combination treatments of drugs with specific kinase inhibitors. The heatmap shows the correlation coefficients of p-site abundances and drug sensitivity across the NCI60 panel (significant Pearson correlations are labeled with S; Benjamini-Hochberg corrected $P < 0.05$). For example, the abundance of the activity-inhibiting p-site S186 on CHEK1 is positively correlated with Artesunate sensitivity (zoom in panel; n=60 cell lines; cell lines are colored by tissue of origin as in Figure 1), which suggests synergy between Artesunate and CHEK1 inhibitors. Source data are provided as a Source Data file.

SUPPLEMENTARY REFERENCES

1. Gholami AM, *et al.* Global proteome analysis of the NCI-60 cell line panel. *Cell Rep* **4**, 609-620 (2013).

2. Frejno M, *et al.* Pharmacoproteomic characterisation of human colon and rectal cancer. *Mol Syst Biol* **13**, 951 (2017).

3. Scherer WF, Syverton JT, Gey GO. Studies on the propagation in vitro of poliomyelitis viruses. IV. Viral multiplication in a stable strain of human malignant epithelial cells (strain HeLa) derived from an epidermoid carcinoma of the cervix. *J Exp Med* **97**, 695-710 (1953).

4. Schneider U, Schwenk HU, Bornkamm G. Characterization of EBV-genome negative "null" and "T" cell lines derived from children with acute lymphoblastic leukemia and leukemic transformed non-Hodgkin lymphoma. *Int J Cancer* **19**, 621-626 (1977).

5. Ruprecht B, Koch H, Domasinska P, Frejno M, Kuster B, Lemeer S. Optimized Enrichment of Phosphoproteomes by Fe-IMAC Column Chromatography. *Methods Mol Biol* **1550**, 47-60 (2017).

6. Ruprecht B, Koch H, Medard G, Mundt M, Kuster B, Lemeer S. Comprehensive and reproducible phosphopeptide enrichment using iron immobilized metal ion affinity chromatography (Fe-IMAC) columns. *Mol Cell Proteomics* **14**, 205-215 (2015).

7. Ruprecht B, Zecha J, Zolg DP, Kuster B. High pH Reversed-Phase Micro-Columns for Simple, Sensitive, and Efficient Fractionation of Proteome and (TMT labeled) Phosphoproteome Digests. *Methods Mol Biol* **1550**, 83-98 (2017).

8. Rappsilber J, Ishihama Y, Mann M. Stop and go extraction tips for matrix-assisted laser desorption/ionization, nanoelectrospray, and LC/MS sample pretreatment in proteomics. *Anal Chem* **75**, 663-670 (2003).

9. Marx H, *et al.* A large synthetic peptide and phosphopeptide reference library for mass spectrometry-based proteomics. *Nat Biotechnol* **31**, 557-564 (2013).

10. Hahne H, *et al.* DMSO enhances electrospray response, boosting sensitivity of proteomic experiments. *Nat Methods* **10**, 989-991 (2013).

11. Klaeger S, *et al.* The target landscape of clinical kinase drugs. *Science* **358**, (2017).

12. McCarty KS, Jr., *et al.* Use of a monoclonal anti-estrogen receptor antibody in the immunohistochemical evaluation of human tumors. *Cancer Res* **46**, 4244s-4248s (1986).

13. Corso J, *et al.* Elucidation of tonic and activated B-cell receptor signaling in Burkitt's lymphoma provides insights into regulation of cell survival. *Proc Natl Acad Sci U S A* **113**, 5688-5693 (2016).

14. Schneider C, *et al.* SAMHD1 is a biomarker for cytarabine response and a therapeutic target in acute myeloid leukemia. *Nat Med* **23**, 250-255 (2017).

15. Schmidt T, *et al.* ProteomicsDB. *Nucleic Acids Res* **46**, D1271-D1281 (2018).

16. Tyanova S, Temu T, Cox J. The MaxQuant computational platform for mass spectrometry-based shotgun proteomics. *Nat Protoc* **11**, 2301-2319 (2016).

17. Schwanhausser B, *et al.* Global quantification of mammalian gene expression control. *Nature* **473**, 337-342 (2011).

18. Webb-Robertson BJ, *et al.* Review, evaluation, and discussion of the challenges of missing value imputation for mass spectrometry-based label-free global proteomics. *J Proteome Res* **14**, 1993-2001 (2015).

19. Medard G, *et al.* Optimized chemical proteomics assay for kinase inhibitor profiling. *J Proteome Res* **14**, 1574-1586 (2015).

20. Reinhold WC, *et al.* CellMiner: a web-based suite of genomic and pharmacologic tools to explore transcript and drug patterns in the NCI-60 cell line set. *Cancer Res* **72**, 3499-3511 (2012).

21. Rees MG, *et al.* Correlating chemical sensitivity and basal gene expression reveals mechanism of action. *Nat Chem Biol* **12**, 109-116 (2016).

22. Garnett MJ, *et al.* Systematic identification of genomic markers of drug sensitivity in cancer cells. *Nature* **483**, 570-575 (2012).

23. Barretina J, *et al.* The Cancer Cell Line Encyclopedia enables predictive modelling of anticancer drug sensitivity. *Nature* **483**, 603-607 (2012).

24. Medico E, *et al.* The molecular landscape of colorectal cancer cell lines unveils clinically actionable kinase targets. *Nat Commun* **6**, 7002 (2015).

25. Bracht K, Nicholls AM, Liu Y, Bodmer WF. 5-Fluorouracil response in a large panel of colorectal cancer cell lines is associated with mismatch repair deficiency. *Br J Cancer* **103**, 340-346 (2010).

26. Tong P, *et al.* drexplorer: A tool to explore dose-response relationships and drug-drug interactions. *Bioinformatics* **31**, 1692-1694 (2015).

27. Loewe S. The problem of synergism and antagonism of combined drugs. *Arzneimittelforschung* **3**, 285-290 (1953).

28. Chou TC, Talalay P. Quantitative analysis of dose-effect relationships: the combined effects of multiple drugs or enzyme inhibitors. *Adv Enzyme Regul* **22**, 27-55 (1984).

29. Martin-Betancor K, Ritz C, Fernandez-Pinas F, Leganes F, Rodea-Palomares I. Defining an additivity framework for mixture research in inducible whole-cell biosensors. *Sci Rep* **5**, 17200 (2015).

30. Zhang B, Horvath S. A general framework for weighted gene co-expression network analysis. *Stat Appl Genet Mol Biol* **4**, Article17 (2005).

31. Langfelder P, Zhang B, Horvath S. Defining clusters from a hierarchical cluster tree: the Dynamic Tree Cut package for R. *Bioinformatics* **24**, 719-720 (2008).

32. Ruepp A, *et al.* CORUM: the comprehensive resource of mammalian protein complexes--2009. *Nucleic Acids Res* **38**, D497-501 (2010).

33. The Gene Ontology C. Expansion of the Gene Ontology knowledgebase and resources. *Nucleic Acids Res* **45**, D331-D338 (2017).

34. Kanehisa M, Furumichi M, Tanabe M, Sato Y, Morishima K. KEGG: new perspectives on genomes, pathways, diseases and drugs. *Nucleic Acids Res* **45**, D353-D361 (2017).

35. Fabregat A, *et al.* The Reactome Pathway Knowledgebase. *Nucleic Acids Res* **46**, D649-D655 (2018).

36. Hanzelmann S, Castelo R, Guinney J. GSVA: gene set variation analysis for microarray and RNA-seq data. *BMC Bioinformatics* **14**, 7 (2013).

37. Ritchie ME, *et al.* limma powers differential expression analyses for RNA-sequencing and microarray studies. *Nucleic Acids Res* **43**, e47 (2015).

38. Benjamini Y, Hochberg Y. Controlling the False Discovery Rate: A Practical and Powerful Approach to Multiple Testing. *Journal of the Royal Statistical Society Series B (Methodological)* **57**, 289-300 (1995).

39. Liberzon A, Subramanian A, Pinchback R, Thorvaldsdottir H, Tamayo P, Mesirov JP. Molecular signatures database (MSigDB) 3.0. *Bioinformatics* **27**, 1739-1740 (2011).

40. Lee E, Chuang HY, Kim JW, Ideker T, Lee D. Inferring pathway activity toward precise disease classification. *PLoS Comput Biol* **4**, e1000217 (2008).

41. Lachmann A, Ma'ayan A. KEA: kinase enrichment analysis. *Bioinformatics* **25**, 684-686 (2009).

42. Dinkel H, *et al.* Phospho.ELM: a database of phosphorylation sites--update 2011. *Nucleic Acids Res* **39**, D261-267 (2011).

43. Hu J, Rho HS, Newman RH, Zhang J, Zhu H, Qian J. PhosphoNetworks: a database for human phosphorylation networks. *Bioinformatics* **30**, 141-142 (2014).

44. Hornbeck PV, Zhang B, Murray B, Kornhauser JM, Latham V, Skrzypek E. PhosphoSitePlus, 2014: mutations, PTMs and recalibrations. *Nucleic Acids Res* **43**, D512-520 (2015).

45. Perfetto L, *et al.* SIGNOR: a database of causal relationships between biological entities. *Nucleic Acids Res* **44**, D548-554 (2016).

46. The UniProt C. UniProt: the universal protein knowledgebase. *Nucleic Acids Res* **45**, D158-D169 (2017).

47. Han J, *et al.* ESEA: Discovering the Dysregulated Pathways based on Edge Set Enrichment Analysis. *Sci Rep* **5**, 13044 (2015).

48. Kanehisa M, Goto S. KEGG: kyoto encyclopedia of genes and genomes. *Nucleic Acids Res* **28**, 27-30 (2000).

49. Schaefer CF, *et al*. PID: the Pathway Interaction Database. *Nucleic Acids Res* **37**, D674-679 (2009).

50. Elkon R, *et al*. SPIKE--a database, visualization and analysis tool of cellular signaling pathways. *BMC Bioinformatics* **9**, 110 (2008).

51. Romero P, Wagg J, Green ML, Kaiser D, Krummenacker M, Karp PD. Computational prediction of human metabolic pathways from the complete human genome. *Genome Biol* **6**, R2 (2005).

52. Mi H, *et al.* Protocol Update for large-scale genome and gene function analysis with the PANTHER classification system (v.14.0). *Nat Protoc* **14**, 703-721 (2019).

53. Cleveland WS. LOWESS: A Program for Smoothing Scatterplots by Robust Locally Weighted Regression *The American Statistician* **35**, 54 (1981).

54. Zou H, Hastie T. Regularization and variable selection via theelastic net. *Journal of Royal Statistical Society B* **67**, 301-320 (2005).

55. Friedmann J, Hastie T, Tibshirani R. Regularization Paths for Generalized Linear Models via Coordinate Descent. *Journal of Statistical Software* **33**, (2010).

56. Szklarczyk D, *et al.* STRING v10: protein-protein interaction networks, integrated over the tree of life. *Nucleic Acids Res* **43**, D447-452 (2015).

57. Liaw A, Wiener M. Classification and regression by randomforest. *R News* **2**, 18-22 (2002).

58. Karaman İ, Nørskov NP, Yde CC, Hedemann MS, Bach Knudsen KE, Kohler A. Sparse multi-block PLSR for biomarker discovery when integrating data from LC–MS and NMR metabolomics. *Metabolomics* **11**, 367-379 (2014).
