# Supplementary material for: Proteome activity landscapes of tumor cell lines determine drug responses
Source: Nat Commun. 2020 Jul 20;11:3639. doi: 10.1038/s41467-020-17336-9 (PMC7371697; doi:10.1038/s41467-020-17336-9)
Supplement: Supplementary file 3 — Description of Additional Supplementary Files [file 41467_2020_17336_MOESM3_ESM.docx]

Description of Additional Supplementary Files

Supplementary Data 1. Related to Figure 1 – Cell line and drug sensitivity information

Supplementary Data 2. Related to Figure 1 – Phosphosite information

Supplementary Data 3. Related to Figure 1 – Protein information

Supplementary Data 4. Related to Figure 1E and Supplementary Figure 2 – Enrichment analysis of

functional associations

Supplementary Data 5. Related to Figure 2 and Supplementary Figure 3 – Integrated kinase substrate

database

Supplementary Data 6. Related to Figure 2E & F and Supplementary Figure 3C – Functional

associations

Supplementary Data 7. Related to Figure 3 & 4F-H and Supplementary Figure 4 – Outlier analysis and

5FU signatures

Supplementary Data 8. Related to Figure 5and 6A-B – SMBPLSR analysis

Supplementary Data 9. Related to Figure 6F and 7C & F – Cox proportional-hazards models of pPGR

& AK1 and AK1 MRM analysis
