# Supplementary material for: Proteome activity landscapes of tumor cell lines determine drug responses
Source: Nat Commun. 2020 Jul 20;11:3639. doi: 10.1038/s41467-020-17336-9 (PMC7371697; doi:10.1038/s41467-020-17336-9)
Supplement: Supplementary file 13 — Reporting Summary [file 41467_2020_17336_MOESM13_ESM.pdf]

nature research

Corresponding author(s): Bernhard Kuster

Last updated by author(s): Jun 16, 2020

Reporting Summary

Nature Research wishes to improve the reproducibility of the work that we publish. This form provides structure for consistency and transparency in reporting. For further information on Nature Research policies, see Authors & Referees and the Editorial Policy Checklist .

Statistics

For all statistical analyses, confirm that the following items are present in the figure legend, table legend, main text, or Methods section.

n/a	Confirmed	
☐	☒	The exact sample size (*n*) for each experimental group/condition, given as a discrete number and unit of measurement
☐	☒	A statement on whether measurements were taken from distinct samples or whether the same sample was measured repeatedly
☐	☒	The statistical test(s) used AND whether they are one- or two-sided<br>*Only common tests should be described solely by name; describe more complex techniques in the Methods section.*
☐	☒	A description of all covariates tested
☐	☒	A description of any assumptions or corrections, such as tests of normality and adjustment for multiple comparisons
☐	☒	A full description of the statistical parameters including central tendency (e.g. means) or other basic estimates (e.g. regression coefficient) AND variation (e.g. standard deviation) or associated estimates of uncertainty (e.g. confidence intervals)
☐	☒	For null hypothesis testing, the test statistic (e.g. *F*, *t*, *r*) with confidence intervals, effect sizes, degrees of freedom and *P* value noted<br>*Give P values as exact values whenever suitable.*
☒	☐	For Bayesian analysis, information on the choice of priors and Markov chain Monte Carlo settings
☒	☐	For hierarchical and complex designs, identification of the appropriate level for tests and full reporting of outcomes
☐	☒	Estimates of effect sizes (e.g. Cohen's *d*, Pearson's *r*), indicating how they were calculated

Our web collection on statistics for biologists contains articles on many of the points above.

Software and code

Policy information about availability of computer code

Data collection	Detailed information is available in the manuscript.
Data analysis	Detailed information is available in the manuscript.

For manuscripts utilizing custom algorithms or software that are central to the research but not yet described in published literature, software must be made available to editors/reviewers. We strongly encourage code deposition in a community repository (e.g. GitHub). See the Nature Research guidelines for submitting code & software for further information.

Data

Policy information about availability of data

All manuscripts must include a data availability statement. This statement should provide the following information, where applicable:

- Accession codes, unique identifiers, or web links for publicly available datasets
- A list of figures that have associated raw data
- A description of any restrictions on data availability

Annotated spectra were uploaded to ProteomicsDB. Additionally, all raw mass spectrometry files and MaxQuant result files are available via PRIDE and ProteomeXchange with the accession code PXD013615. Our previously published proteomics data on the CRC65 cell line panel has the accession code PXD005354.

Field-specific reporting

Please select the one below that is the best fit for your research. If you are not sure, read the appropriate sections before making your selection.

☒ Life sciences ☐ Behavioural & social sciences ☐ Ecological, evolutionary & environmental sciences

Life sciences study design

All studies must disclose on these points even when the disclosure is negative.

Sample size	n/a
Data exclusions	It is worth noting that we excluded CoCM-1 cells from all analyses because of a consistently lower amount of identified proteins and p-sites (Supplementary Figure 1E).
Replication	n/a
Randomization	n/a
Blinding	n/a

Reporting for specific materials, systems and methods

We require information from authors about some types of materials, experimental systems and methods used in many studies. Here, indicate whether each material, system or method listed is relevant to your study. If you are not sure if a list item applies to your research, read the appropriate section before selecting a response.

Materials & experimental systems

n/a	Involved in the study	
☐	☒	Antibodies
☐	☒	Eukaryotic cell lines
☒	☐	Palaeontology
☒	☐	Animals and other organisms
☒	☐	Human research participants
☒	☐	Clinical data

Methods

n/a	Involved in the study	
☒	☐	ChIP-seq
☒	☐	Flow cytometry
☒	☐	MRI-based neuroimaging

Antibodies

Antibodies used	PGR antibody (#PI633C01, clone 16, DCS)<br>PGR_pS162 antibody (#GTX80351-50, Lot Nr. 821604961, Biozol)<br>AK1 antibody (#OAAB17548, Aviva Systems Biology, USA)<br>GAPDH antibody (#sc-365062, Santa Cruz Biotechnology, USA)
Validation	PI633C01 is an antibody used routinely in clinical practice. GTX80351-50 was tested in Humans. It cross-reacts with phosphoserine 163 in mouse progesterone receptor. OAAB17548 was validated in our manuscript by western blot. sc-365062 was extensively validated elsewhere (see e.g. https://www.ncbi.nlm.nih.gov/pmc/articles/PMC6629652/)

Eukaryotic cell lines

Policy information about cell lines

Cell line source(s)	See Supplementary Table 1.
Authentication	None of the cell lines were authenticated.
Mycoplasma contamination	Cell lines were not tested for mycoplasma contamination.
Commonly misidentified lines<br>(See ICLAC register)	See Supplementary Table 1. The NCI60 cell line panel is well known and there is more information available on MDA-MB-435, U251, SNB-19 and NCI/ADR-RES here: https://dtp.cancer.gov/discovery_development/nci-60/cell_list.htm
